# Exploring the Chemical Dynamics of Phenylethynyl Radical (C_6_H_5_CC; X^2^A_1_) Reactions with Allene (H_2_CCCH_2_; X^1^A_1_) and Methylacetylene (CH_3_CCH; X^1^A_1_)

**DOI:** 10.1021/acs.jpca.3c03077

**Published:** 2023-07-04

**Authors:** Shane J. Goettl, Zhenghai Yang, Siegfried Kollotzek, Dababrata Paul, Ralf I. Kaiser, Ankit Somani, Adrian Portela-Gonzalez, Wolfram Sander, Anatoliy A. Nikolayev, Valeriy N. Azyazov, Alexander M. Mebel

**Affiliations:** † Department of Chemistry, University of Hawaii at Ma̅noa, Honolulu, Hawaii 96822, United States; ‡ Lehrstuhl für Organische Chemie II, Ruhr-Universität Bochum, Bochum 44801, Germany; § Samara National Research University, Samara 443086, Russia; ∥ Lebedev Physical Institute, Samara 443011, Russia; ⊥ Department of Chemistry and Biochemistry, Florida International University, Miami, Florida 33199, United States

## Abstract

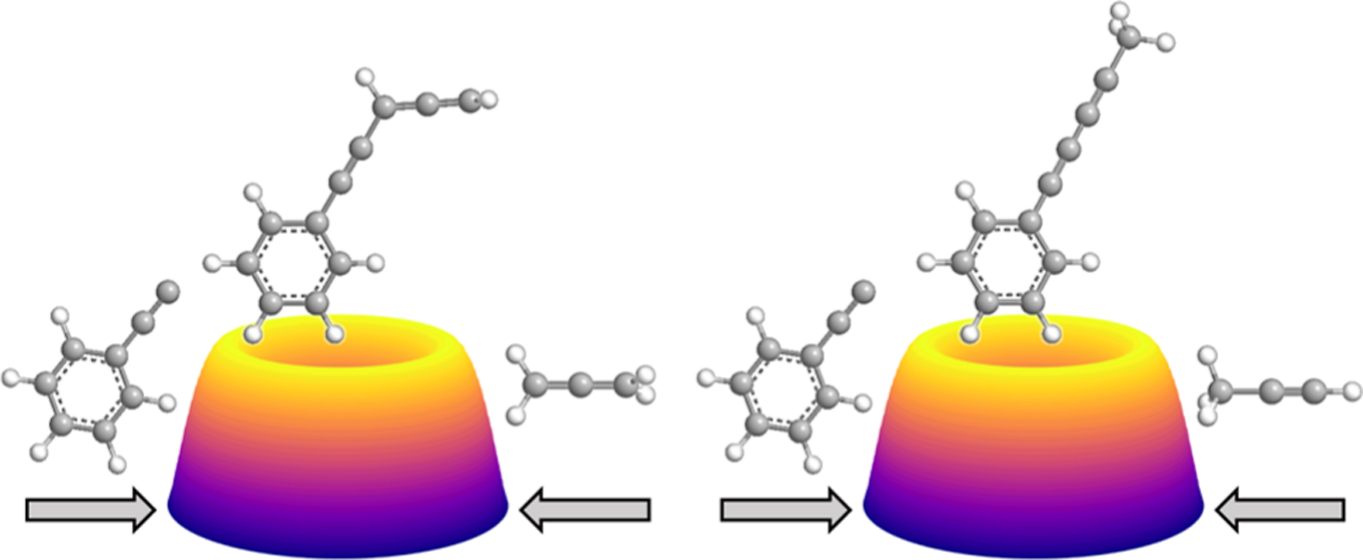

The bimolecular gas-phase reactions of the phenylethynyl radical (C_6_H_5_CC, X^2^A_1_) with allene (H_2_CCCH_2_), allene-*d*_4_ (D_2_CCCD_2_), and methylacetylene (CH_3_CCH) were studied under single-collision conditions utilizing the crossed molecular beams technique and merged with electronic structure and statistical calculations. The phenylethynyl radical was found to add without an entrance barrier to the C1 carbon of the allene and methylacetylene reactants, resulting in doublet C_11_H_9_ collision complexes with lifetimes longer than their rotational periods. These intermediates underwent unimolecular decomposition via atomic hydrogen loss through tight exit transition states in facile radical addition—hydrogen atom elimination mechanisms forming predominantly 3,4-pentadien-1-yn-1-ylbenzene (C_6_H_5_CCCHCCH_2_) and 1-phenyl-1,3-pentadiyne (C_6_H_5_CCCCCH_3_) in overall exoergic reactions (−110 kJ mol^–1^ and −130 kJ mol^–1^) for the phenylethynyl–allene and phenylethynyl–methylacetylene systems, respectively. These barrierless reaction mechanisms mirror those of the ethynyl radical (C_2_H, X^2^Σ^+^) with allene and methylacetylene forming predominantly ethynylallene (HCCCHCCH_2_) and methyldiacetylene (HCCCCCH_3_), respectively, suggesting that in the aforementioned reactions the phenyl group acts as a spectator. These molecular mass growth processes are accessible in low-temperature environments such as cold molecular clouds (TMC-1) or Saturn’s moon Titan, efficiently incorporating a benzene ring into unsaturated hydrocarbons.

## 1 Introduction

The formation mechanisms of polycyclic aromatic hydrocarbons (PAHs) along with their unsaturated precursors have received considerable attention by the astrochemistry and combustion science communities.^1−3^ Here, PAHs are classified as reaction intermediates and fundamental molecular building blocks in molecular mass growth processes leading ultimately to soot particles (combustion flames) and carbonaceous nanoparticles (circumstellar and interstellar grains).^4,5^ Particular interest has been devoted to the propargyl radical (H_2_CCCH, X^2^B_1_), which represents a prototype of a resonantly stabilized free radical (RSFR) and the most thermodynamically stable C_3_H_3_ isomer.^6^ Recently detected in the cold Taurus Molecular Cloud (TMC-1),^7^ bimolecular propargyl–propargyl radical reactions lead to the formation of the aromatic phenyl radical (C_6_H_5_), while a stabilization of the reaction intermediate(s) accesses benzene (C_6_H_6_) along with its 1,5-hexadiyne, fulvene, and 2-ethynyl-1,3-butadiene isomers.^8−10^ Consequently, the propargyl radical plays a major role in astrochemical^11−13^ and combustion^14−18^ models as a potential precursor for bottom-up synthetic pathways to PAHs and carbonaceous nanoparticles (soot, interstellar grains). However, reactions of the propargyl radical with closed-shell hydrocarbons, e.g., acetylene (C_2_H_2_) and benzene (C_6_H_6_), involve entrance barriers to addition typically in the range of 50–60 kJ mol^–1^.^19−21^ These entrance barriers limit propargyl radical reactions with closed-shell hydrocarbons to high-temperature environments like circumstellar envelopes of carbon stars and planetary nebulae as their descendants. So far, the reaction of tricarbon (C_3_)—formally a carbene—with the propargyl radical represents the only barrierless and exoergic pathway of the propargyl radical with a technically closed-shell ‘organic’ reactant leading to triacetylene (HCCCCCCH) and its high-energy isomer ethynylbutatrienylidene (HCCCHCC).^11,22^

**Figure 1:**
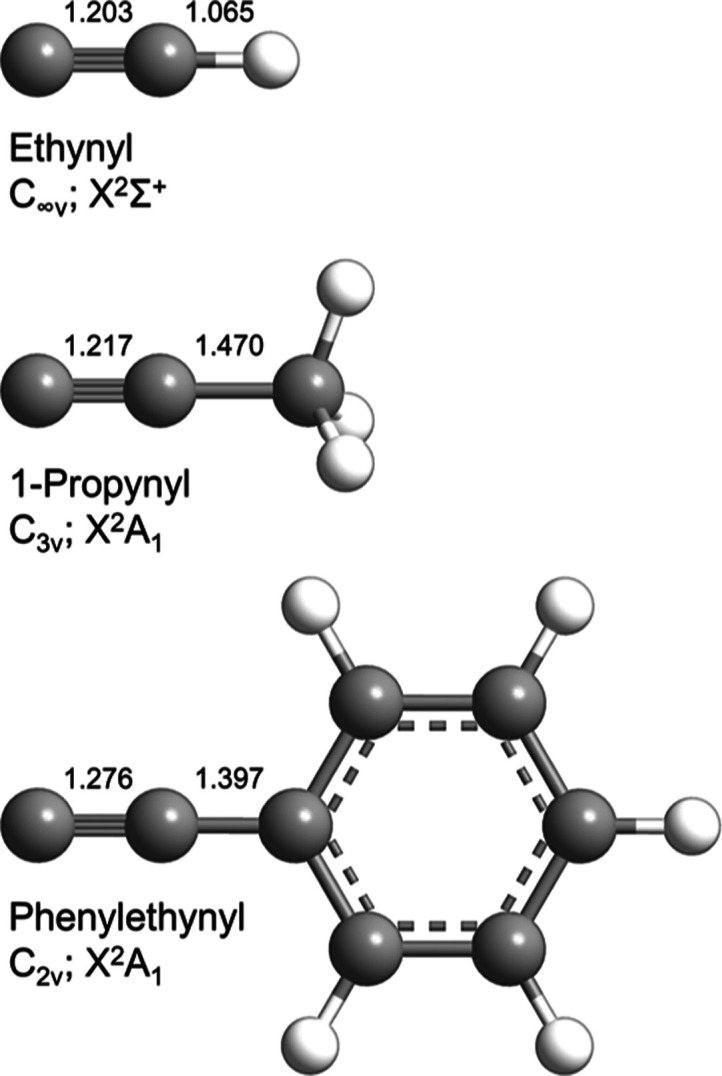
Structures of three substituted acetylenic radicals: ethynyl (**1**), 1-propynyl (**2**), and phenylethynyl (**3**). Internuclear distances are shown in Ångström (Å). Carbon atoms are color coded in gray, while hydrogen atoms are colored in white.

On the other hand, the 1-propynyl radical (CH_3_CC, X^2^A_1_)—a non-resonant free-radical isomer of C_3_H_3_ (Figure 1)—lies 168 kJ mol^–1^ higher in energy than propargyl^6^ and has recently been demonstrated to add without an entrance barrier to the closed-shell hydrocarbons acetylene,^23^ ethylene (C_2_H_4_),^24^ methylacetylene (CH_3_CCH), allene (H_2_CCCH_2_),^25^ diacetylene (C_4_H_2_),^26^ 1,3-butadiene (C_4_H_6_),^27^ and benzene (C_6_H_6_).^28^ The barrierless nature of bimolecular encounters in these systems implies that the 1-propynyl radical—in strong contrast to the propargyl radical isomer—can initiate reactions forming highly unsaturated hydrocarbons—among them polyacetylenes such as methyldiacetylene^23,29^ and methyltriacetylene,^26^ as well as aromatics like toluene^27^ and 1-phenyl-1-propyne^28^—in low-temperature environments like cold molecular clouds.^30−32^

These molecular mass growth studies of the 1-propynyl radical can be expanded by substituting the methyl group (−CH_3_) with a phenyl group (−C_6_H_5_), giving rise to the isolobal phenylethynyl radical (C_6_H_5_CC, X^2^A_1_). Previous studies classified this species as a π radical due to the antisymmetric mixing of the π_z_ orbital of the ethynyl group with the E_1a_ π orbital of the phenyl moiety;^33,34^ however, a recent investigation indicates that phenylethynyl represents rather a σ-type radical with an X^2^A_1_ electronic ground state in analogy to the ethynyl (C_2_H) and 1-propynyl (CH_3_CC) radicals. The phenylethynyl radical was initially suggested to form via photodissociation of (2-iodoethynyl)benzene;^35,36^ this production route was verified in low-temperature argon matrices.^33^ A gas chromatography–mass spectrometry (GC–MS) analysis of the dimethyl disulfide-scavenged decomposition products of phenylacetylene (C_6_H_5_CCH) via flow reactor pyrolysis at 1300 K by Hofmann et al.^37^ detected phenylethynyl radicals along with their *o*-, *m*-, and *p*-ethynylphenyl (C_6_H_4_CCH) isomers. Phenylethynyl radicals were also shown to form via hydrogen abstraction from reactions of hydroxyl (OH)^38^ and ethynyl (C_2_H)^39^ from phenylacetylene. Recently, crossed molecular beams studies on the reaction of dicarbon (C_2_, X^1^Σ_g_^+^/a^3^Π_u_) with benzene produced phenylethynyl radicals under single-collision conditions, suggesting that dicarbon reacts as a pseudohalogen with benzene.^40^ The aforementioned findings resulted in the incorporation of the phenylethynyl radical into a combustion model by Hamadi et al., where the pyrolysis of benzene (C_6_H_6_) in the presence of acetylene (C_2_H_2_) and vinylacetylene (C_4_H_4_) at a temperature range of 1100–1800 K leads to the formation of phenylacetylene (C_6_H_5_CCH) coupled with atomic hydrogen loss. Subsequently, abstraction of the acetylenic hydrogen produces the phenylethynyl radical. These species can react with vinylacetylene, and this reaction was postulated to produce naphthalene (C_10_H_8_) plus atomic hydrogen.^41^ However, while previous investigations report multiple formation pathways of the phenylethynyl radical, detailed molecular mass growth processes commencing with phenylethynyl have not been explored experimentally to date.

Herein, we report on the bimolecular gas-phase reactions of the phenylethynyl radical (C_6_H_5_CC, X^2^A_1_) with allene (H_2_CCCH_2_), allene-*d*_*4*_ (D_2_CCCD_2_), and methylacetylene (CH_3_CCH) under single-collision conditions by exploiting the crossed molecular beams technique. By combining the experimental results with electronic structure calculations, we reveal the predominant formation of 3,4-pentadien-1-yn-1-ylbenzene (C_6_H_5_CCCHCCH_2_) and 1-phenyl-1,3-pentadiyne (C_6_H_5_CCCCCH_3_) in the allene and methylacetylene reactions, respectively, via C_11_H_9_ reaction intermediates. These pathways are initiated by the addition of a phenylethynyl radical center to the C1 carbon of the C_3_H_4_ isomers without an entrance barrier featuring long-lived intermediate(s) before unimolecular decomposition via atomic hydrogen loss. These barrierless processes serve as a nontraditional, hitherto neglected route for the gas-phase preparation of highly unsaturated hydrocarbons through the incorporation of a phenyl group as precursors to more complex PAHs leading eventually to carbonaceous nanostructures in low-temperature environments such as cold molecular clouds.

## 2 Methods

### 2.1 Experimental Methods

The reactions of the phenylethynyl radical (C_6_H_5_CC) with allene (H_2_CCCH_2_, 98%, Organic Technologies), allene-*d*_4_ (D_2_CCCD_2_, 98% D atom, CDN Isotopes), and methylacetylene (CH_3_CCCH, 99%, Organic Technologies) were conducted by utilizing a crossed molecular beams machine.^42^ The apparatus consists of a 2.3 m^3^ stainless-steel chamber, which houses the primary (phenylethynyl) and secondary (allene, allene-*d*_4_, methylacetylene) source chambers as well as a triply differentially pumped quadrupole mass spectrometric detector held at ultrahigh vacuum (UHV, 6 × 10^–12^ Torr) conditions. The latter is rotatable within the plane defined by both molecular beams. The phenylethynyl (C_6_H_5_CC) radical beam was produced by photodissociation of neon-seeded (2-iodoethynyl)benzene (C_6_H_5_CCI, Supporting Information). The precursor was held in a stainless-steel bubbler at room temperature and purified with multiple freeze–pump–thaw cycles. The complete bubbler assembly was then placed inside the primary source chamber to reduce the distance between the bubbler and pulsed valve, thus decreasing sample loss due to sticking to the tubing and preventing clogs. The (2-iodoethynyl)benzene precursor was seeded at a fraction of 0.5% in neon (Ne, 99.9999%, Matheson) at a backing pressure of 500 Torr and fed through a Proch-Trickl pulsed valve^43^ operating a piezoelectric disc translator (Physik Instrumente, P-286.23) at 120 Hz, −450 V, opening times of 80 μs, and a primary source chamber pressure of 5 × 10^–5^ Torr. The neon–precursor gas mixture exited the pulsed valve located at a distance of 16 ± 1 mm from a stainless-steel skimmer. A pulsed 193 nm, 20 mJ output from an ArF (Nova Gas, MIX-78-44-6000) excimer laser (Coherent, COMPex 110) was focused (2 × 3 mm^2^) 1 mm downstream of the pulsed valve nozzle, intersected the supersonic beam of (2-iodoethynyl)benzene/neon, and generated phenylethynyl radicals (C_6_H_5_CC). The supersonic beam then passed through the skimmer and was velocity selected by a chopper wheel located 11.6 ± 0.6 mm downstream of the skimmer. On-axis (Θ = 0°) characterization of the primary beam at an electron impact ionization energy of 26 eV provided a peak velocity *v*_p_ of 862 ± 19 m s^–1^ and speed ratio *S* of 17.7 ± 1.3 for the phenylethynyl radical. In the secondary source chamber, a pulsed allene beam (*v*_p_ = 800 ± 10 m s^–1^, *S* = 12.0 ± 0.4) operating at 120 Hz at a backing pressure of 550 Torr and pulsed valve voltage of −350 V led to a secondary source chamber pressure of 5 × 10^–5^ Torr at pulsed valve opening times of 80 μs. The allene beam passed through a skimmer located 18.0 ± 0.1 mm downstream of the secondary pulsed valve nozzle before crossing perpendicularly with the phenylethynyl radical beam resulting in a collision energy *E*_c_ of 19.8 ± 0.7 kJ mol^–1^ and center-of-mass (CM) angle Θ_CM_ of 20.9 ± 0.6°. Experiments conducted with allene-*d*_4_ (*v*_p_ = 790 ± 10 m s^–1^, *S* = 12.0 ± 0.4) and methylacetylene (*v*_p_ = 800 ± 10 m s^–1^, *S* = 12.0 ± 0.4) provided collision energies of 21.0 ± 0.7 and 19.8 ± 0.7 kJ mol^–1^ as well as CM angles of 22.5 ± 0.7 and 20.9 ± 0.6°, respectively (Table 1). Note that both the primary and secondary beams pass through an oxygen-free high-conductivity (OFHC) copper cold shield located 8.1 ± 0.1 mm upstream of the interaction region; this shield was cooled to 10 K via a cold head (CTI Cryogenics, model 1020) to reduce background counts in the detector from straight-through molecules.

**Table 1:** Peak Velocities (*v*_p_) and Speed Ratios (*S*) for the Phenylethynyl Radical (C_6_H_5_CC), Allene (H_2_CCCH_2_), Allene-*d*_*4*_ (D_2_CCCD_2_), and Methylacetylene (CH_3_CCH) Beams As Well As the Corresponding Collision Energies (*E*_c_) and Center-of-Mass Angles (Θ_CM_) for Each Reactive Scattering Experiment

beam	*v*_p_ (m s^–1^)	*S*	*E*_c_ (kJ mol^–1^)	Θ_CM_ (°)
C_6_H_5_CC (X^2^A_1_)	862 ± 19	17.7 ± 1.3		
H_2_CCCH_2_ (X^1^A_1_)	800 ± 10	12.0 ± 0.4	19.8 ± 0.7	20.9 ± 0.6
D_2_CCCD_2_ (X^1^A_1_)	790 ± 10	12.0 ± 0.4	21.0 ± 0.7	22.5 ± 0.7
CH_3_CCH (X^1^A_1_)	800 ± 10	12.0 ± 0.4	19.8 ± 0.7	20.9 ± 0.6

Products formed from the reactions of phenylethynyl radicals with allene, allene-*d*_*4*_, and methylacetylene were detected after electron impact ionization by exploiting a triply differentially pumped quadrupole mass spectrometric detector operating in the time-of-flight (TOF) mode. The detector consists of three regions: regions I and II reduce the gas load from the main chamber, while region III houses a modified Brink-type^44^ electron impact ionizer operating at 80 eV during reactive scattering experiments, which is surrounded by a liquid nitrogen-cooled jacket. Standard pressures in region III reach 6 × 10^–12^ Torr, while incorporating a 4 K cold shield can reduce pressures down to 8 × 10^–13^ Torr.^45^ Neutral species ionized in region III were filtered according to mass-to-charge ratio (*m*/*z*) by a quadrupole mass spectrometer (Extrel, 150QC) operating with a 1.2 MHz oscillator. The ions were accelerated onto an aluminum-coated high-voltage (−22.5 kV) target, causing a cascade of secondary electrons directed toward an aluminum-coated organic scintillator (BC-418, Saint Gobain). The photons were collected by a photo-multiplier tube (Burle, model 8850) operated at −1.35 kV. The output signal was discriminated (Advanced Research Instruments, Model F-100TD) at 1.6 mV and recorded by a multichannel scaler (Stanford Research Systems, SRS 40) to obtain TOF spectra.

**Figure 2:**
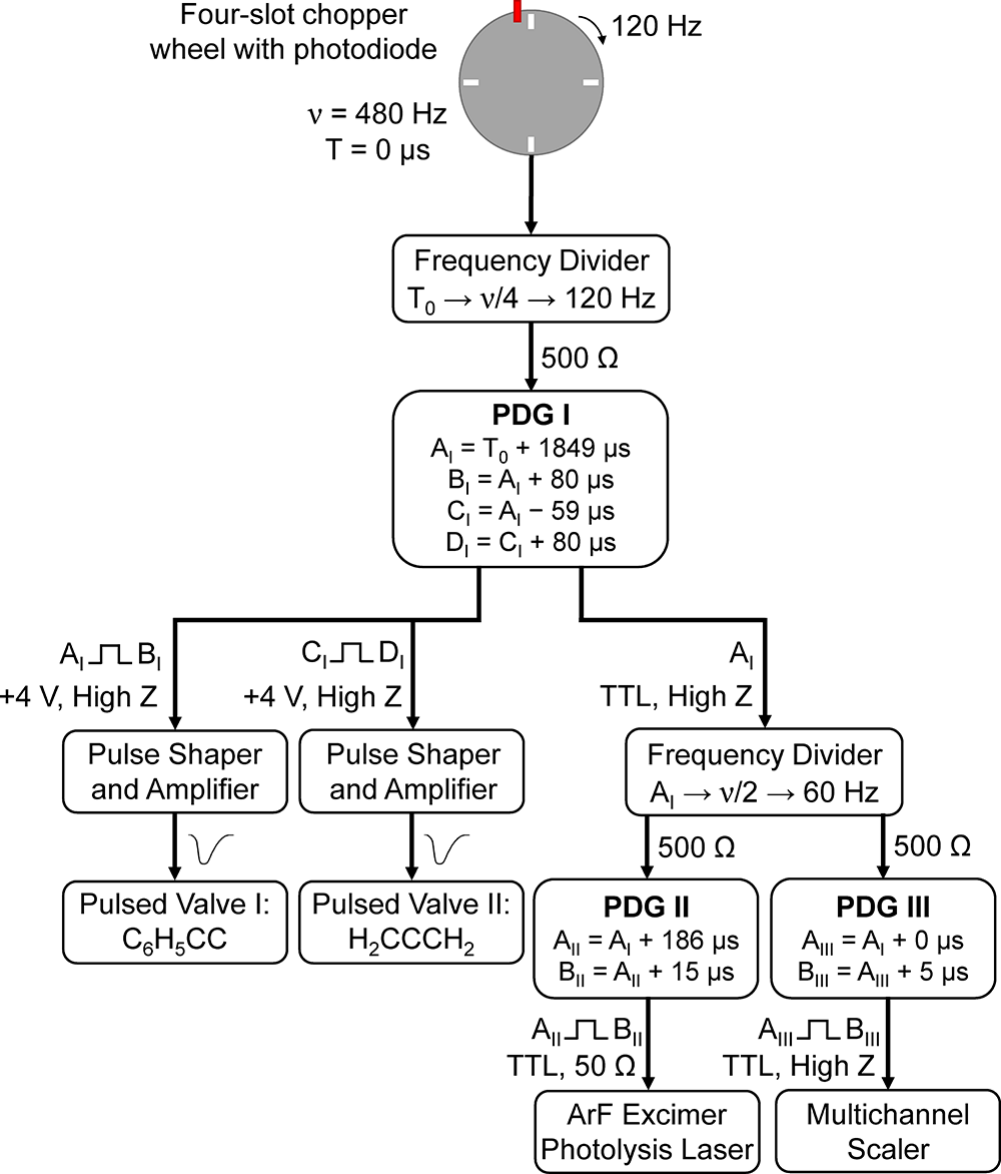
Pulse sequence for the crossed molecular beams machine for the phenylethynyl (C_6_H_5_CC)–allene (H_2_CCCH_2_) reaction.

Due to the pulsed nature of the experiment, a precise time sequence was required (Figure 2). A pulse from an infrared photodiode located at the top of a 17.0 ± 0.1 cm diameter, four-slot (0.76 ± 0.01 mm) chopper wheel rotating at 120 Hz served as the time zero (*T*_0_ = 0 μs) and hence the trigger for the pulse sequence. In detail, the 480 Hz signal from the photodiode was sent through a ν/4 frequency divider, and the resulting 120 Hz signal was relayed to a pulse/delay generator (PDG I, Stanford Research Systems, DG 535). For the phenylethynyl–allene reaction, the PDG I outputs (+4 V, high impedance) AB (*A*_I_ = *T*_0_ + 1849 μs, *B*_I_ = *A*_I_ + 80 μs) and CD (*C*_I_ = *A*_I_ – 59 μs, *D*_I_ = *C*_I_ + 80 μs) were sent through a pulse shaper and pulse amplifier (E-421, Physik Instrumente) and were received by the primary and secondary pulsed valves, respectively. The output from PDG I A (TTL, high impedance) was halved to 60 Hz and sent to PDGs II and III for laser-on minus laser-off background subtraction. The AB output (TTL, 50 Ω) of PDG II (*A*_II_ = *A*_I_ + 186 μs, *B*_II_ = *A*_II_ + 15 μs) triggered the excimer laser, while the AB output (TTL, high impedance) of PDG III (*A*_III_ = *A*_I_ + 0 μs, *B*_III_ = *A*_III_ + 5 μs) triggered the multichannel scaler. The delays for the phenylethynyl–allene-*d*_4_ reaction were as follows: PDG I AB (*A*_I_ = *T*_0_ + 1849 μs, *B*_I_ = *A*_I_ + 80 μs) and CD (*C*_I_ = *A*_I_ – 59 μs, *D*_I_ = *C*_I_ + 80 μs); PDG II AB (*A*_II_ = *A*_I_ + 184 μs, B_II_ = A_II_ + 15 μs); PDG III AB (*A*_III_ = *A*_I_ + 0 μs, *B*_III_ = *A*_III_ + 5 μs). The delays for the phenylethynyl–methylacetylene reaction were as follows: PDG I AB (*A*_I_ = *T*_0_ + 1849 μs, *B*_I_ = *A*_I_ + 80 μs) and CD (*C*_I_ = *A*_I_ – 59 μs, *D*_I_ = *C*_I_ + 80 μs); PDG II AB (*A*_II_ = *A*_I_ + 184 μs, *B*_II_ = *A*_II_ + 15 μs); PDG III AB (*A*_III_ = *A*_I_ + 0 μs, *B*_III_ = *A*_III_ + 5 μs).

Up to 4 × 10^6^ angularly resolved TOFs were obtained at angles between 11° ≤ Θ ≤ 36° with respect to the primary beam (Θ = 0°). These spectra were integrated and normalized to the CM angle to obtain a laboratory angular distribution. To extract the reaction dynamics herein, the data were transformed from the laboratory to the CM reference frame exploiting a forward convolution routine.^46,47^ This generated user-defined product translational energy (*P*(*E*_T_)) and angular (*T*(θ)) flux distributions, which were refined iteratively until a reasonable fit of the data was achieved. The CM functions also define the product flux contour map, which reveals the differential reactive cross section, *I*(*u*,θ) ≈ *P*(*u*) × *T*(θ), as intensity with respect to the angle θ and the CM velocity *u.*(48) This flux contour map contains all information on the scattering process and can be seen as an image of the reaction.

### 2.2 Computational Methods

Geometries of the reactants, products, intermediates, and transition states on the C_11_H_9_ potential energy surface (PES) involved in the reactions of the phenylethynyl radical with allene and methylacetylene were optimized at the hybrid density functional ωB97X-D/6-311G(d,p) level of theory^49^ with vibrational frequencies computed using the same method. Energies of reactants, products, and various C_11_H_9_ species were consequently rectified by using single-point calculations within G3(MP2,CC) model chemistry,^50−52^ with the final energies computed as
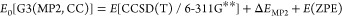
with Δ*E*_MP2_ = *E*[MP2/G3Large] – *E*[MP2/6-311G**] and *E*(ZPE) being the basis set correction and zero-point vibrational energy, respectively. The anticipated accuracy of this computational scheme for relative energies is within 4–8 kJ mol^–1^. The Gaussian 09^53^ and MOLPRO 2010^54^ programs were utilized for the DFT, MP2, and CCSD(T) calculations.

Next, energy-dependent rate constants for various unimolecular reaction steps taking place on the C_11_H_9_ PES following the formation of collision complexes were computed utilizing the Rice–Ramsperger–Kassel–Marcus (RRKM) theory.^55−57^ The internal energy of all C_11_H_9_ species and products was set to be equal to the sum of the collision and chemical activation energies. Here, the latter is obtained as a negative of the relative energy of each species with regard to the separated C_8_H_5_ + C_3_H_4_ reactants. The rate constants were computed using our own in-house code.^25^ The calculations were performed at the zero-pressure limit emulating the crossed molecular beams single-collision conditions. The steady-state approximation along with RRKM rate constants were utilized to assess product branching ratios depending on the reaction collision energy.^25,58^

## 3 Results

### 3.1 Laboratory Frame

**Figure 3:**
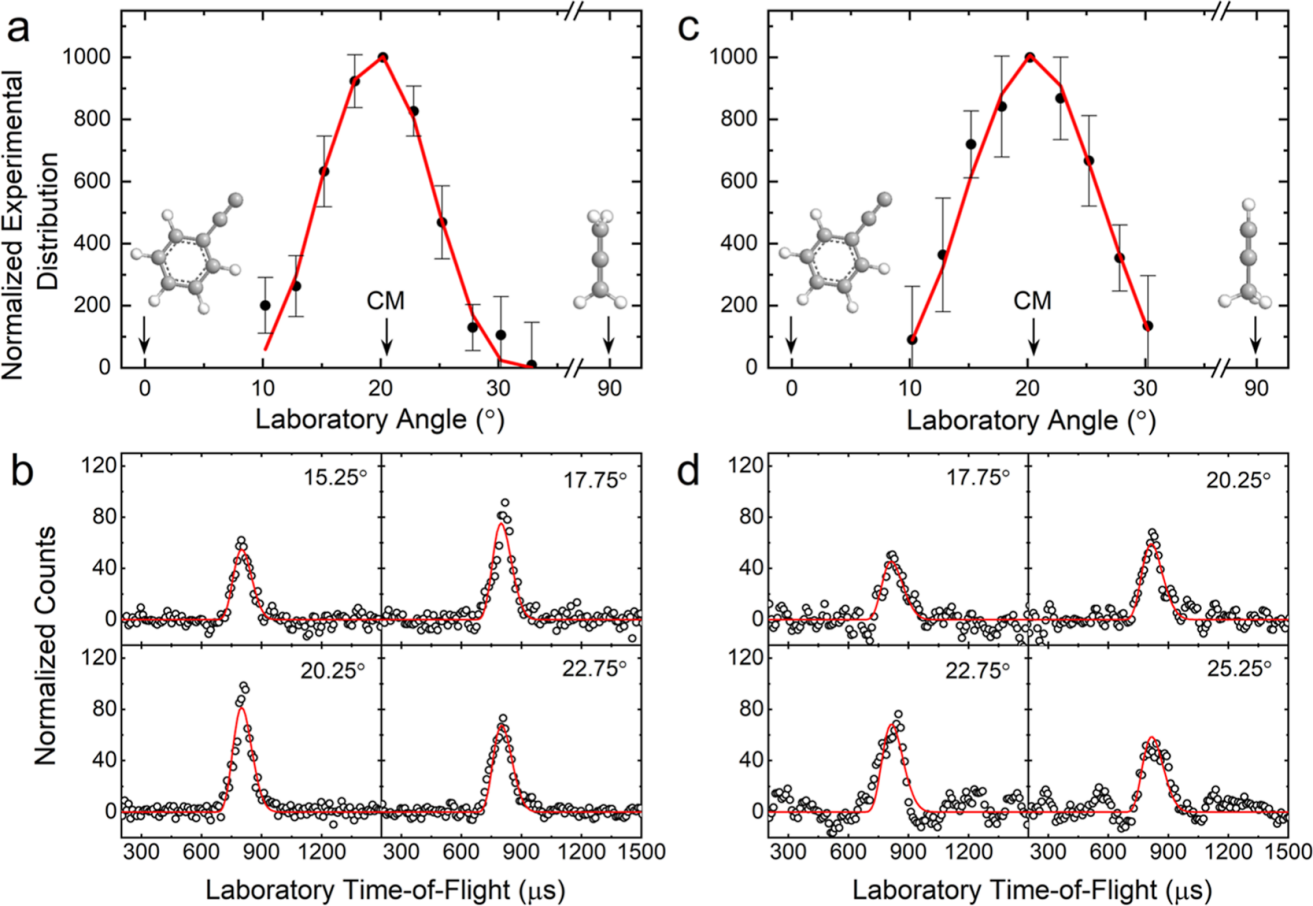
Laboratory angular distributions and time-of-flight (TOF) spectra for the reaction of phenylethynyl (C_6_H_5_CC) with allene (H_2_CCCH_2_) (a,b) and with methylacetylene (CH_3_CCH) (c,d) recorded at *m*/*z* = 140. CM represents the center-of-mass angle, and 0 and 90° define the directions of the phenylethynyl and allene/methylacetylene beams, respectively. The black circles depict the data and red lines the fits. Carbon atoms are color coded in gray, while hydrogen atoms are colored in white.

For the bimolecular reaction of the phenylethynyl radical (C_6_H_5_CC) with allene (H_2_CCCH_2_), reactive scattering signals were observed at *m*/*z* = 140 (C_11_H_8_^+^) and 139 (C_11_H_7_^+^). These TOFs overlap after scaling. This finding indicates that the signal at *m*/*z* = 139 originates from dissociative electron impact ionization of the neutral C_11_H_8_ parent molecule (reaction 1). Signal for the C_11_H_9_ adduct at *m*/*z* = 141 was not detected. Since ion counts were of similar intensity for *m*/*z* = 140 and 139 ((0.87 ± 0.05):1), TOFs were collected for the parent ion at *m*/*z* = 140 (Figure 3b), which were relatively narrow ranging from about 700 to 900 μs. The laboratory angular distribution features a forward-backward symmetry with respect to the CM angle of 20.9 ± 0.6°. This result indicates indirect reaction dynamics through C_11_H_9_ intermediate(s) leading to the C_11_H_8_ isomer(s) plus atomic hydrogen. In order to elucidate the position of the atomic hydrogen loss, i.e., from the phenyl ring and/or from the allene reactant, the phenylethynyl (C_6_H_5_CC) reaction with allene-*d*_4_ (D_2_CCCD_2_) was studied (reaction 2). TOFs were collected at *m*/*z* = 144 (C_11_H_4_D_4_^+^) and 143 (C_11_H_5_D_3_^+^) at the center-of-mass angle of 23°. Signal was observed at both masses with counts at *m*/*z* = 144 at a level of 16 ± 6% compared to *m*/*z* = 143 (Figure S1). Both TOFs overlap after scaling, indicating that ion counts at *m*/*z* = 143 can be attributed to the formation of C_11_H_5_D_3_ isomer(s) plus atomic deuterium, whereas detected counts at *m*/*z* = 144 likely originate from the naturally occurring ^13^CC_10_H_5_D_3_^+^. Thus, the phenylethynyl–allene-*d*_4_ experiment reveals an emission of a deuterium atom, indicating that C_11_H_8_ product(s) are formed in the unlabeled reaction through H loss from the allene reactant.

Finally, ion counts for the reaction of the phenylethynyl radical (C_6_H_5_CC) with methylacetylene (CH_3_CCH) were observed at *m*/*z* = 140 (C_11_H_8_^+^) (reaction 3). TOFs were also searched for at *m*/*z* = 141 (C_11_H_9_^+^, ^13^CC_10_H_8_^+^) and 139 (C_11_H_7_^+^), but no ion counts were perceivable above the noise level. Figure 3d shows the TOFs collected at *m*/*z* = 140, which also depict signal from about 700–900 μs. The laboratory angular distribution (Figure 3c) exhibits forward–backward symmetry with respect to the center-of-mass angle of 20.9 ± 0.6° and features intensity over a range of 20°. These findings mimic those in the phenylethynyl–allene reaction, indicating indirect reaction dynamics through C_11_H_9_ intermediate(s) leading to C_11_H_8_ product(s) plus atomic hydrogen. No isotopically labeled methylacetylene experiments were conducted due to low signal intensity.
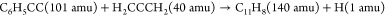
1
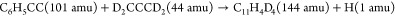
2a
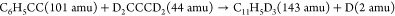
2b
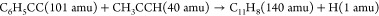
3

### 3.2 Center-of-Mass Frame

**Figure 4:**
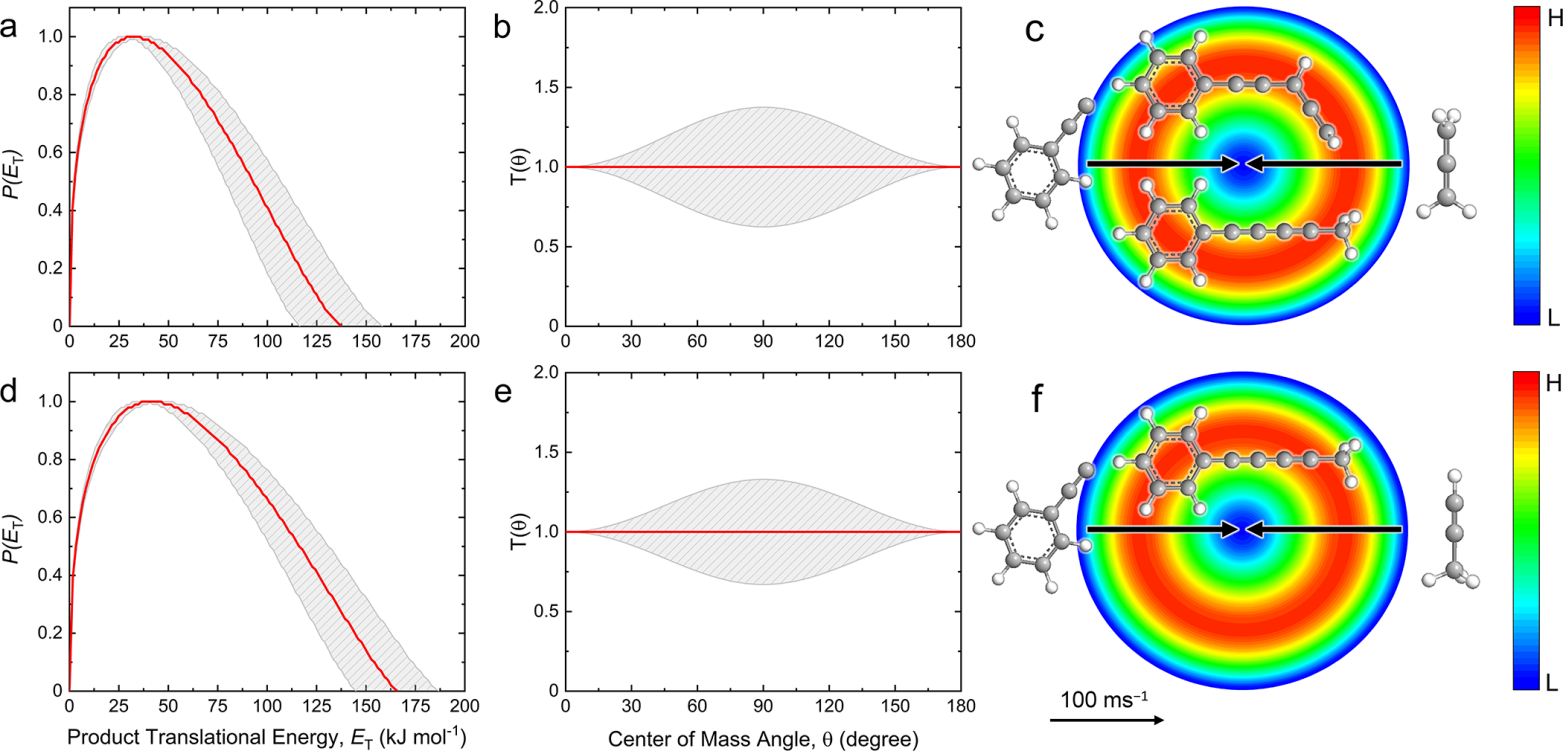
CM translational energy (a,d) and angular (b,e) flux distributions, as well as the associated flux contour maps (c,f) leading to the formation of C_11_H_8_ isomers plus atomic hydrogen in the reaction of phenylethynyl (C_6_H_5_CC) with allene (H_2_CCCH_2_) (a–c) and with methylacetylene (CH_3_CCH) (d–f). Red lines define the best-fit functions, while shaded areas provide the error limits. The flux contour map represents the intensity of the reactively scattered products as a function of product velocity (*u*) and scattering angle (θ), and the color bar indicates flux gradient from high (H) to low (L) intensity. Carbon atoms are color coded in gray, while hydrogen atoms are colored in white.

Following the observation of C_11_H_8_ product(s) through hydrogen atom loss from the reactions of phenylethynyl with allene (reaction 1) and methylacetylene (reaction 3), we now elucidate the underlying reaction dynamics. For both the allene and methylacetylene systems, the TOFs and laboratory angular distribution could be fit with a single channel corresponding to C_11_H_8_ plus atomic hydrogen. The best-fitting CM functions, *P*(*E*_T_) and *T*(θ), are shown in Figure 4. Inspecting first the *P*(*E*_T_), the phenylethynyl–allene system (Figure 4a) exhibits a maximum translational energy *E*_max_ of 137 ± 21 kJ mol^–1^. The relationship *E*_max_ = *E*_c_ – Δ_r_*G* can be exploited to recover reaction energies for products born without internal excitation; thus, a reaction energy of −117 ± 22 kJ mol^–1^ is derived. Additionally, the *P*(*E*_T_) peaks at 32 kJ mol^–1^, indicating a tight exit transition state upon decomposition of the C_11_H_9_ intermediate(s) to the final products. The phenylethynyl–methylacetylene system (Figure 4d) features a higher *E*_max_ of 166 ± 21 kJ mol^–1^ corresponding to a reaction energy of −146 ± 22 kJ mol^–1^. The *P*(*E*_T_) exhibits a maximum at 42 kJ mol^–1^, also signifying that this system has a tight exit transition state to form the C_11_H_8_ product(s) plus atomic hydrogen.

Additional information can be gained by analyzing the *T*(θ) for both reactions. In the phenylethynyl–allene system (Figure 4b), the *T*(θ) depicts nonzero intensity along the entire angular range suggesting indirect reaction dynamics leading to C_11_H_8_ product(s) through C_11_H_9_ intermediate(s). The forward–backward symmetry is indicative of a lifetime of the intermediate(s) longer than the rotational periods. These findings are nearly identical to those found in the phenylethynyl–methylacetylene system (Figure 4e), in which a best-fit isotropic (flat) distribution over all angles implies indirect reaction dynamics through activated C_11_H_9_ complex(es) with a lifetime longer than the rotational period. These results are reflected in the flux contour maps for both systems (Figure 4c,f).

## 4 Discussion

**Figure 5:**
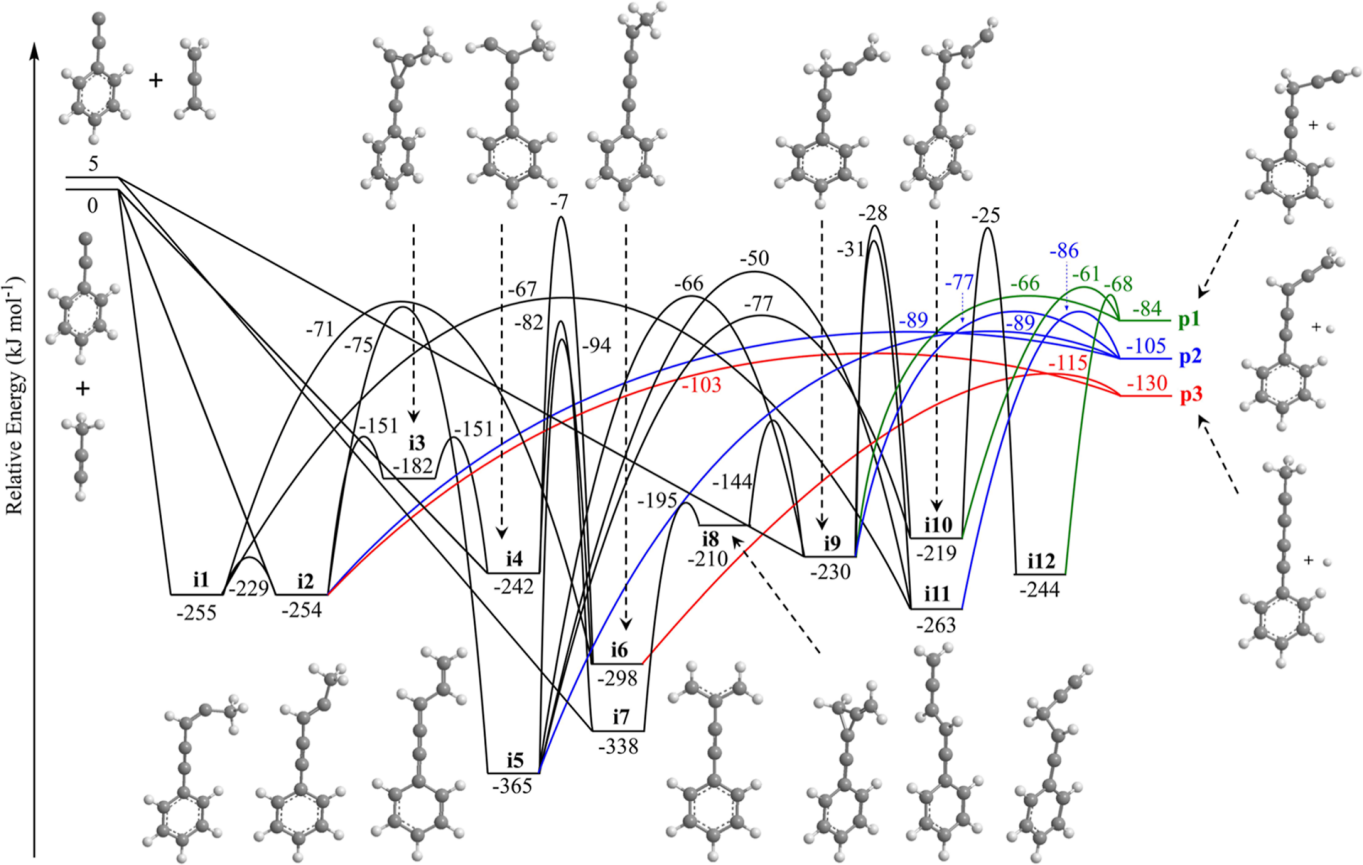
Calculated potential energy surface for the hydrogen atom loss products of the reaction of phenylethynyl (C_6_H_5_CC) with allene (H_2_CCCH_2_) and with methylacetylene (CH_3_CCH) at the G3(MP2,CC)//ωB97X-D/6-311G(d,p) level. Energies are in units of kJ mol^–1^, and colored pathways denote exit channels to the products. The full potential energy surface is shown in Figure S2. Carbon atoms are color coded in gray, while hydrogen atoms are colored in white.

With the detection of C_11_H_8_ isomer(s) from atomic hydrogen loss via the reactions of phenylethynyl (C_6_H_5_CC) with allene (H_2_CCCH_2_) and methylacetylene (CH_3_CCH) through long-lived C_11_H_9_ intermediate(s), we now merge these results with electronic structure calculations to determine reaction pathways and product isomers. The full PES with six products (**p1–p6**), 12 intermediates (**i1–i12**), and 31 transition states featuring both the phenylethynyl–allene and phenylethynyl–methylacetylene reactions is compiled in Figure S2, whereas a reduced PES featuring the atomic hydrogen loss products (**p1–p3**) is shown in Figure 5.

### 4.1 Phenylethynyl–Allene System

For the phenylethynyl–allene system, the experimentally derived reaction energy of −117 ± 22 kJ mol^–1^ matches two of the calculated products, 3,4-pentadien-1-yn-1-ylbenzene (**p2**, −110 ± 5 kJ mol^–1^) and 1-phenyl-1,3-pentadiyne (**p3**, −135 ± 5 kJ mol^–1^); however, contributions from the 1,4-pentadiyn-1-ylbenzene (**p1**, −89 ± 5 kJ mol^–1^) isomer may be hidden within the lower energy portion of the *P*(*E*_T_). Therefore, this isomer cannot be discounted for at the present stage. First, we discuss the pathways to **p1**. The reaction is initiated with the addition of the phenylethynyl radical with the radical center to either the central (C2) or terminal (C1/C3) carbon of allene, forming intermediates **i7** and/or **i9**, respectively, without an entrance barrier. Product **p1** can be formed from a simple addition–elimination pathway in which the initial collision complex **i9** is formed followed by hydrogen atom loss from the terminal carbon of the side chain over a 164 kJ mol^–1^ barrier involving a tight exit transition state. Additional pathways to **p1** involve hydrogen migration from **i9** to **i10** via a 202 kJ mol^–1^ barrier followed by hydrogen atom loss over a tight exit transition state; further, a hydrogen migration from **i10** to **i12** may precede a unimolecular decomposition of **i12** via hydrogen atom loss. Collision complex **i7** can isomerize via phenylethynyl migration through a three-membered ring intermediate to **i8** followed by ring opening to **i9**, which then leads to **p1** through the same routes as mentioned above. Considering the unfavorable barriers to isomerization to **i10** and **i12**, the preferred pathway to **p1** likely obeys the following straightforward sequence: reactants **→ i9 → p1**. This is reinforced by considering calculated rate constants at a collision energy of 20 kJ mol^–1^ (Table S1) under the assumption of complete energy randomization in the reaction intermediate, which indicate that the **i9 → p1** step (*k*(*E*) = 7.76 × 10^4^ s^–1^) is faster than any of the competing isomerization pathways to **i10** or **i12**.

Product **p2** can be produced through an addition–elimination pathway via **i9** with a tight exit transition state located 28 kJ mol^–1^ above the separated products. Multiple alternate routes to **p2** exist and involve the rearrangement of intermediate **i9** by hydrogen atom shift through a 199 kJ mol^–1^ barrier to **i11** prior to a hydrogen atom loss forming **p2** as well as additional hydrogen atom migration–hydrogen atom loss pathways involving **i9 → i5 → p2** and **i9 → i5 → i2 → p2**. Collision complex **i7** may also lead to **p2** after isomerization to **i9** as detailed above, while **i7** can also undergo hydrogen atom migration to **i4**. From here, pathways to **p2** include **i4 → i6 → i5 → p2**, **i4 → i6 → i1 → i2 → p2** and **i4 → i3 → i2 → p2**. Taking into account the high barriers of isomerization as well as the computed rate constants (Table S1), product **p2** is likely formed from the addition–elimination pathway via reactants **→ i9 → p2**. Therefore, intermediate **i9** represents the central intermediate to both products **p1** and **p2**.

Unlike **p1** and **p2**, product **p3** cannot be formed from an addition–elimination pathway; therefore, isomerization steps of the collision complexes are required. There are two exit channels to **p3**, leading from **i2** and **i6** through tight exit transition states. After barrierless addition of the radical reactant, collision complexes **i9** and **i7** may isomerize to **i2** and **i6**. For intermediate **i9**, this involves a series of hydrogen migrations (**i9 → i10/i11 → i5 → i6/i2**), as well as a bond rotation from **i1** to **i2** after hydrogen shift from **i11**. For **i7**, isomerization can lead to **i4**, which can either rearrange over a high barrier of 235 kJ mol^–1^ to **i6** or may undergo a facile ring closure to **i3** followed by ring opening to **i2**. Intermediates **i9** and **i7** can easily interconvert through **i8** over barriers of 86 kJ mol^–1^ and 143 kJ mol^–1^, respectively, and hence can follow the same routes as discussed above. Taking into account the isomerization steps from the calculated rate constants (Table S1) for each reaction sequence, the routes **i9 → i10 → i5 → i6 → p3** and **i7 → i8 → i9 → i10 → i5 → i6 → p3** are likely the preferred pathways to **p3** in the phenylethynyl–allene reaction.

Statistical branching ratios for the atomic hydrogen loss products calculated for the phenylethynyl–allene reaction at various collision energies are shown in Table 2. As our experimental collision energy was 19.8 ± 0.7 kJ mol^–1^, the branching ratios calculated at 20 kJ mol^–1^ were used for a comparison. Starting from the initial collision complex of **i7** or **i9** yields the same result since the isomerization between **i7** and **i9** through **i8** is very fast compared to alternative steps. Products **p1–p3** were calculated to form at levels of 34.9, 62.0, and 3.1%, respectively; this reflects the PES, where both **p1** and **p2** can be formed from a facile addition–elimination pathway, while **p3** can only be produced after extensive isomerization.

**Table 2:** Statistical Branching Ratios (%) for the Hydrogen Atom Loss Pathways for the Reaction of Phenylethynyl (C_6_H_5_CC) with Allene (H_2_CCCH_2_) and with Methylacetylene (CH_3_CCH) at Different Collision Energies (*E*_C_*,* kJ mol^–1^)

Phenylethynyl + allene										
	initial intermediate *i7*					initial intermediate *i9*				
*E*_C_	**0**	**10**	**20**	**30**	**40**	**0**	**10**	**20**	**30**	**40**
**p1**	27.3	31.2	34.9	38.2	41.1	27.3	31.2	34.9	38.2	41.1
**p2**	65.7	64.9	62.0	59.2	56.7	67.8	64.9	62.0	59.2	56.7
**p3**	5.0	3.9	3.1	2.6	2.2	4.9	3.9	3.1	2.6	2.2

phenylethynyl + methylacetylene										
	initial intermediates ***i1*/*i2***					initial intermediate ***i4***				
*E*_C_	**0**	**10**	**20**	**30**	**40**	**0**	**10**	**20**	**30**	**40**
**p1**	0.4	0.3	0.4	0.3	0.6	0.4	0.3	0.3	0.7	0.8
**p2**	12.1	13.4	14.9	16.1	17.4	12.1	13.6	15.1	16.2	17.3
**p3**	87.5	86.3	84.7	83.6	82.0	87.5	86.1	84.6	83.1	81.9

To summarize, the experimentally derived reaction energy from the phenylethynyl–allene system indicates the formation of **p2** and/or **p3**, though it cannot be discriminated which, if not both, are formed since they lie within the error bars of the reaction energy. Utilizing the computed PES and branching ratios, products **p1** and **p2** are favored through their one-step addition–elimination pathways at levels of 34.9 and 62.0%, respectively. Product **p3** requires multiple isomerization prior to unimolecular decomposition via hydrogen atom loss and thus is likely a minor product as confirmed via statistical calculations revealing its fraction of only 3.1%.

### 4.2 Phenylethynyl–Methylacetylene System

The experimental reaction energy of −146 ± 22 kJ mol^–1^ for the phenylethynyl–methylacetylene system matches that calculated for **p3** (−130 ± 5 kJ mol^–1^), while products **p1** (−84 kJ ± 5 mol^–1^) and **p2** (−105 ± 5 kJ mol^–1^) are higher in energy and thus could be veiled within the *P*(*E*_T_). The phenylethynyl radical center can add to either the C1 or C2 carbon of methylacetylene without barrier yielding **i1/i2**—separated by a low barrier for the rotation around the carbon–carbon single bond of 26 kJ mol^–1^ with respect to **i1** or **i4**, respectively. First, all pathways to **p1** involve extensive isomerization. Intermediate **i1** can rearrange to **i11** and onward to **i9** through two successive hydrogen atom migrations before atomic hydrogen loss over an exit barrier of 18 kJ mol^–1^ with respect to the separated **p1** + H products. Intermediates **i10** and **i12**, accessible through additional H shifts starting from **i9**, also lead to **p1** via H loss over tight exit transition states. Additional pathways to **p1** follow **i4 → i7 → i8 → i9 → p1** and **i2 → i5 → i9/i10 → p1**. Calculated rate constants for the phenylethynyl–methylacetylene system (Table S2) suggest that the most likely pathway to **p1** obeys the following sequence: reactants **→ i4 → i7 → i8 → i9 → p1**.

Product **p2** can be formed through a simple addition–elimination mechanism from **i2** via atomic hydrogen loss through an exit barrier of 16 kJ mol^–1^ with respect to the separated products. Intermediate **i2** can also isomerize via hydrogen atom migration to **i5,** which undergoes unimolecular decomposition to **p2**. Additional exit channels to **p2** lead from **i9** and **i11**, the former of which can be produced through hydrogen atom migration from **i5** or from the **i4 → i7 → i8 → i9** sequence discussed above, while the latter can be formed from hydrogen atom shift from **i1**, **i5**, or **i9**. Based on the barrier heights and necessary isomerization steps for these routes, the addition–elimination mechanism, reactants **→ i2 → p2**, is favored. The calculated rate constants strengthen this argument, as the **i2 → p2** exit channel has a larger rate constant (*k*(*E*) = 1.80 × 10^5^ s^–1^) than any of the competing exit channels leading to **p2**.

Like for **p2**, product **p3** can be produced through an addition–elimination mechanism, reactants **→ i2 → p3**, via an exit barrier with the transition state located 27 kJ mol^–1^ above the separated products. The only other exit channel to **p3** leads over a barrier of 183 kJ mol^–1^ from **i6** (15 kJ mol^–1^ above the separated products), where **i6** could be formed from **i1**, **i4**, and **i5**. It is likely that the pathway reactants **→ i2 → p3** is the preferred pathway to **p3** since this involves no rearrangements between intermediates and the rate constant for **i2 → p3** is 2 orders of magnitude higher than for **i6 → p3**.

Branching ratios for the atomic hydrogen loss products of the phenylethynyl–methylacetylene reaction were also computed and are shown in Table 2. The branching ratios starting from intermediates **i1** or **i2** are essentially identical since they are separated only by a low barrier to rotation. Likewise, the branching ratios from the initial intermediate **i4** are very similar to those calculated, starting from **i1/i2** since **i2** and **i4** are connected through **i3** via a facile phenylethynyl shift. Products **p1–p3** were calculated to form at levels of 0.4/0.3%, 14.9/15.1%, and 84.7/84.6%, respectively, for the initial collision complexes **i1/i2** and **i4**. This is dictated by the PES where **p2** and **p3** can be formed through an addition–elimination process, while the system has to undergo multiple isomerization steps before the formation of **p1**.

To summarize the phenylethynyl–methylacetylene system, the experimentally derived reaction energy of −146 ± 22 kJ mol^–1^ matches the calculated reaction energy of −130 kJ mol^–1^ for **p3**, providing strong evidence for its formation; however, both **p1** and **p2** could be hidden within the lower energy portion of the *P*(*E*_T_) and thus are still possible products. The calculated branching ratios corroborate the formation of **p3** as the major product, which is reflected by the simple one-step addition–elimination mechanism on the computed PES. The **i2 → p2** pathway features a higher exit barrier and smaller rate constant than the **i2 → p3** path, while **p1** requires extensive intermediate rearrangements to be reached; therefore, products **p2** and **p1** are likely only minor products in the phenylethynyl–methylacetylene reaction.

## 5 Conclusions

The crossed molecular beams technique was utilized to explore the reactions of phenylethynyl radicals (C_6_H_5_CC) with allene (H_2_CCCH_2_) and methylacetylene (CH_3_CCH) in the gas phase under single-collision conditions. The combined experimental and computational results indicate that the reactions were initiated with barrierless addition, accessing the C_11_H_9_ doublet PES through long-lived intermediate(s) before subsequent hydrogen atom loss to 3,4-pentadien-1-yn-1-ylbenzene (**p2**) and/or 1-phenyl-1,3-pentadiyne (**p3**) for the phenylethynyl–allene system and 1-phenyl-1,3-pentadiyne (**p3**) for the phenylethynyl–methylacetylene system in overall exoergic reactions. Additionally, our statistical analysis suggests that **p2** (62%) is the major product of the phenylethynyl–allene reaction at a collision energy of 20 kJ mol^–1^, whereas **p3** (3%) provides only a minor contribution. The statistics for the phenylethynyl–methylacetylene system suggest that **p3** (85%) is the major product. Together with the PES analysis, these findings highlight the addition–elimination pathways through intermediates **i9** and **i2** as the most likely routes upon phenylethynyl addition to allene and methylacetylene, respectively. Experiments utilizing allene-*d*_*4*_ are in agreement, showing that the hydrogen loss occurs within our error limits from the allene moiety.

**Figure 6:**
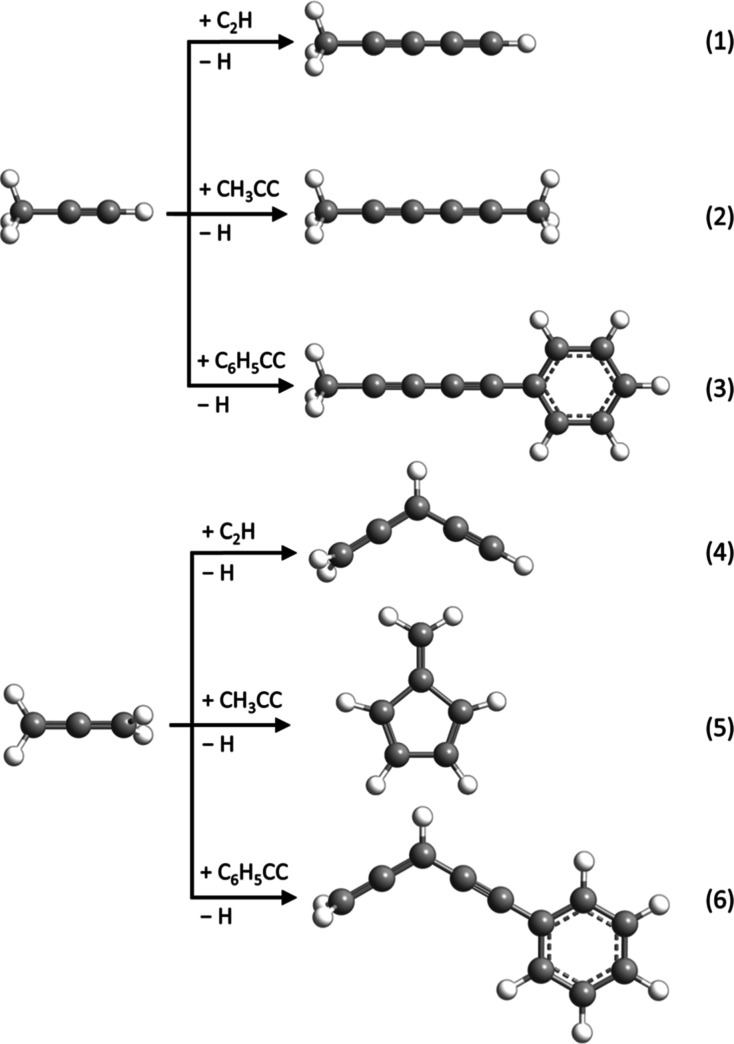
Major product channels from the reactions of methylacetylene (CH_3_CCH) and allene (H_2_CCCH_2_) with ethynyl (C_2_H), 1-propynyl (CH_3_CC), and phenylethynyl (C_6_H_5_CC) radicals. Carbon atoms are color coded in gray, while hydrogen atoms are colored in white.

The results for the reactions of the phenylethynyl radical reactions with allene and methylacetylene can be compared to the isolobal reactions of ethynyl (C_2_H) and 1-propynyl (CH_3_CC) with allene and methylacetylene. Both the ethynyl–methylacetylene^59,60^ and 1-propynyl–methylacetylene^25^ systems follow reaction dynamics similar to the phenylethynyl–methylacetylene system, in which the ethynyl/1-propynyl adds without barrier to the C1 carbon of methylacetylene followed by hydrogen atom loss from the attacked carbon giving the major products methyldiacetylene (**1**) and dimethyldiacetylene (**2**), respectively (Figure 6). On the other hand, the allene reactions do not all follow the same trend. The ethynyl–allene^61^ and phenylethynyl–allene systems both progress through similar addition–elimination mechanisms; however, the 1-propynyl–allene reaction involves several isomerizations eventually forming fulvene (C_5_H_4_CH_2_). This deviation is likely due to low lying vibrational modes of the collision complexes leading to longer lifetimes of the intermediate thus promoting rearrangement to the lower energy fulvene route. Overall, our study on the barrierless and exoergic reactions of phenylethynyl radicals with allene and methylacetylene offers insight on the molecular mass growth of highly unsaturated hydrocarbons possible in low-temperature environments such as TMC-1 and Titan.

## Supporting Information Available

(2-Iodoethynyl)benzene synthesis and characterization, calculated rate constants of the phenylethynyl–allene and phenylethynyl–methylacetylene reactions, full list of branching ratios, CM TOF profiles for the phenylethynyl–allene-*d*_4_ reaction, full PES, NMR spectra of (2-iodoethynyl)benzene, Cartesian coordinates, and IR frequencies (PDF)
