# Supplementary material for: Exploring the Chemical Dynamics of Phenylethynyl Radical (C6H5CC; X2A1) Reactions with Allene (H2CCCH2; X1A1) and Methylacetylene (CH3CCH; X1A1)
Source: J Phys Chem A. 2023 Jul 4;127(27):5723–33. doi: 10.1021/acs.jpca.3c03077 (PMC10350955; doi:10.1021/acs.jpca.3c03077)
Supplement: Supplementary file 1 — jp3c03077_si_001.pdf [file jp3c03077_si_001.pdf]

Supporting Information for

Exploring the Chemical Dynamics of Phenylethynyl Radical (C_6H_5CC; X^2A_1) Reactions with Allene (H_2CCCH_2; X^1A_1) and Methylacetylene (CH_3CCH; X^1A_1)

Shane J. Goettl[a], Zhenghai Yang[a], Siegfried Kollotzek[a,b], Dababrata Paul[a], Ralf I Kaiser*[a]

[a] *Department of Chemistry, University of Hawai'i at Mānoa, Honolulu, Hawai'i 96822, United States*

[b] *Permanent address: Institut für Ionenphysik und Angewandte Physik, Universität Innsbruck, A-6020 Innsbruck, Austria*

Ankit Somani[c], Adrian Portela-Gonzalez[c], Wolfram Sander*[c]

[c] *Lehrstuhl für Organische Chemie II, Ruhr-Universität Bochum, 44801 Bochum, Germany*

Anatoliy A. Nikolayev[d], Valeriy N. Azyazov[e]

[d] *Samara National Research University, Samara 443086, Russia*

[e] *Lebedev Physical Institute, Samara 443011, Russia*

Alexander M. Mebel*[f]

[f] *Department of Chemistry and Biochemistry, Florida International University, Miami, FL 33199, United States*

*Email: ralfk@hawaii.edu, wolfram.sander@rub.de, mebela@fiu.edu

1. Precursor Synthesis and Characterization

The synthesis of the (2-iodoethynyl)benzene precursor was performed analogously to a reported procedure[1] with an increase in the concentration of the starting materials due to the large scale of precursor needed. Regarding the purification, badges performed with less than 10 g of phenylacetylene could be purified by either column chromatography or distillation whereas larger badges (20–50 g) decomposed before affording the desired product and, thus, had to be purified by column chromatography. A solution of phenylacetylene (26.0 mL, 24.2 g, 0.16 mol) in MeOH (300 mL) was cooled to 0 °C and KOH (32.5 g, 0.41 mol) was added. After 20 min of stirring at 0 °C, N-iodosuccinimide (63.1 g, 0.27 mol) was added to the mixture in portions and stirred at 0 °C for 15 min. The cold bath was replaced by a rt water bath and the mixture was stirred for 30 min. Then, Et_2O (1 L) was added and the mixture was extracted with brine (5 x 150 mL), dried over $MgSO_4$, filtered and evaporated to afford a brown oil/liquid. This liquid was purified by column chromatography (SiO_2; hexane) to afford the product as a light-yellow oil (32.5 g, 60%). ^1H NMR (200 MHz, $CDCl_3$): δ = 7.44 (dd, 2H), 7.34 (m, 3H) ppm. ^{13}C NMR (50 MHz, $CDCl_3$): δ = 132.43, 128.92, 128.36, 123.46, 94.26 and 6.48 ppm. GC-MS: m/z 228.1 [M^+] 101.1 [M^+–I]. The characteristic C≡C stretching vibration was found at 2172 cm^{-1} whereas for the starting material it is observed at 2110 cm^{-1}. The provided characterization is in accordance with the literature.[1] ^1H and ^{13}C NMR data were recorded with a Bruker DPX-200 NMR spectrometer referenced towards $CDCl_3$ (7.26 ppm for ^1H NMR and 77.16 ppm for ^{13}C NMR). The GC-MS data were recorded with an Agilent 7820A GC spectrometer. IR data were recorded with a Bruker Equinox 55 FT-IR spectrometer.

Table S1. RRKM calculated rate constants ($k(E)$, s^{-1}) of the reaction of phenylethynyl (C_6H_5CC) with allene (H_2CCCH_2) as functions of the internal energy (E, kJ mol^{-1}) of the intermediate states for unimolecular reaction steps at different collision energies (E_C, kJ mol^{-1}).

E_C	0		10		20		30		40	
**Reaction step**	*k(E)*	*E*	*k(E)*	*E*	*k(E)*	*E*	*k(E)*	*E*	*k(E)*	*E*
i9 – p2	2.70E+04	234	6.22E+04	244	1.33E+05	254	2.66E+05	264	5.03E+05	274
i9 – p1	1.12E+04	234	3.11E+04	244	7.76E+04	254	1.78E+05	264	3.80E+05	274
i2 – p3	4.05E+05	259	8.01E+05	269	1.50E+06	279	2.68E+06	289	4.61E+06	299
i2 – p2	5.55E+04	259	1.24E+05	269	2.59E+05	279	5.10E+05	289	9.56E+05	299
i1 – i11	1.35E+02	260	3.11E+02	270	6.70E+02	280	1.36E+03	290	2.65E+03	300
i11 – i1	1.80E+01	268	4.19E+01	278	9.15E+01	288	1.89E+02	298	3.70E+02	308
i7 – i8	8.11E+06	343	1.13E+07	353	1.54E+07	363	2.07E+07	373	2.74E+07	383
i8 – i7	2.64E+12	214	2.80E+12	224	2.96E+12	234	3.12E+12	244	3.27E+12	254
i1 – i6	2.42E+03	260	5.68E+03	270	1.25E+04	280	2.58E+04	290	5.08E+04	300
i6 – i1	2.58E+01	303	6.59E+01	313	1.57E+02	323	3.52E+02	333	7.43E+02	343
i9 – i8	1.36E+08	234	1.87E+08	244	2.52E+08	254	3.32E+08	264	4.29E+08	274
i8 – i9	6.57E+09	214	8.54E+09	224	1.09E+10	234	1.37E+10	244	1.69E+10	254
i9 – i11	1.02E+00	234	3.77E+00	244	1.22E+01	254	3.56E+01	264	9.44E+01	274
i11 – i9	2.12E-02	268	8.43E-02	278	2.95E-01	288	9.20E-01	298	2.60E+00	308
i9 – i10	1.09E+01	234	4.05E+01	244	1.34E+02	254	4.03E+02	264	1.10E+03	274
i10 – i9	8.33E+00	224	3.02E+01	234	9.81E+01	244	2.87E+02	254	7.68E+02	264
i4 – p4	3.73E+06	247	7.28E+06	257	1.34E+07	267	2.37E+07	277	4.00E+07	287
i4 – i7	2.18E+04	247	4.34E+04	257	8.20E+04	267	1.48E+05	277	2.57E+05	287
i7 – i4	1.23E+00	343	2.95E+00	353	6.67E+00	363	1.42E+01	373	2.90E+01	383
i10 – p1	1.73E+03	224	4.98E+03	234	1.29E+04	244	3.03E+04	254	6.59E+04	264
i10 – p6	1.86E+08	224	2.92E+08	234	4.43E+08	244	6.52E+08	254	9.34E+08	264
i1 – i2	1.12E+12	260	1.21E+12	270	1.29E+12	280	1.37E+12	290	1.45E+12	300
i2 – i1	1.50E+12	259	1.60E+12	269	1.70E+12	279	1.80E+12	289	1.90E+12	299
i4 – i6	5.26E-06	247	1.30E-04	257	1.41E-03	267	9.71E-03	277	4.99E-02	287
i6 – i4	1.33E-08	303	3.70E-07	313	4.50E-06	323	3.46E-05	333	1.97E-04	343
i9 – i5	2.13E+03	234	4.89E+03	244	1.04E+04	254	2.10E+04	264	4.00E+04	274
i5 – i9	1.28E-02	370	3.79E-02	380	1.04E-01	390	2.63E-01	400	6.26E-01	410
i11 – p5	1.05E+07	268	1.85E+07	278	3.13E+07	288	5.09E+07	298	8.03E+07	308
i11 – p2	6.99E+03	268	1.65E+04	278	3.62E+04	288	7.46E+04	298	1.45E+05	308
i6 – p3	1.84E+04	303	3.66E+04	313	6.91E+04	323	1.25E+05	333	2.18E+05	343
i6 – p4	1.16E+06	303	2.16E+06	313	3.88E+06	323	6.68E+06	333	1.11E+07	343
i2 – i5	1.52E+03	259	3.49E+03	269	7.47E+03	279	1.51E+04	289	2.89E+04	299
i5 – i2	4.42E-02	370	1.23E-01	380	3.19E-01	390	7.69E-01	400	1.75E+00	410
i2 – i3	7.42E+07	259	1.05E+08	269	1.45E+08	279	1.96E+08	289	2.61E+08	299
i3 – i2	1.13E+11	187	1.30E+11	197	1.47E+11	207	1.66E+11	217	1.85E+11	227
i4 – i3	2.00E+08	247	2.75E+08	257	3.70E+08	267	4.88E+08	277	6.34E+08	287

i3 – i4	9.65E+10	187	1.11E+11	197	1.26E+11	207	1.41E+11	217	1.58E+11	227
i5 – p2	1.65E+00	370	4.50E+00	380	1.14E+01	390	2.69E+01	400	6.00E+01	410
i11 – i5	2.55E+00	268	7.18E+00	278	1.86E+01	288	4.51E+01	298	1.02E+02	308
i5 – i11	7.40E-04	370	2.49E-03	380	7.67E-03	390	2.19E-02	400	5.80E-02	410
i10 – i5	1.68E+04	224	3.26E+04	234	6.01E+04	244	1.06E+05	254	1.79E+05	264
i5 – i10	6.58E-02	370	1.69E-01	380	4.09E-01	390	9.30E-01	400	2.01E+00	410
i6 – i5	1.00E+03	303	2.04E+03	313	3.95E+03	323	7.32E+03	333	1.31E+04	343
i5 – i6	3.62E+00	370	8.20E+00	380	1.76E+01	390	3.61E+01	400	7.09E+01	410
i10 – i12	5.37E-01	224	1.85E+00	234	5.79E+00	244	1.67E+01	254	4.41E+01	264
i12 – i10	1.10E-01	249	3.99E-01	259	1.32E+00	269	4.01E+00	279	1.11E+01	289
i12 – p5	1.10E+08	249	1.76E+08	259	2.72E+08	269	4.08E+08	279	5.97E+08	289
i12 – p1	8.24E+02	249	2.28E+03	259	5.71E+03	269	1.32E+04	279	2.83E+04	289

Table S2. RRKM calculated rate constants ($k(E)$, s^{-1}) of the reaction of phenylethynyl (C_6H_5CC) with methylacetylene (H_3CCCH) as functions of the internal energy (E, kJ mol^{-1}) of the intermediate states for unimolecular reaction steps at different collision energies (E_C, kJ mol^{-1}).

E_C	0		10		20		30		40	
**Reaction step**	*k(E)*	*E*	*k(E)*	*E*	*k(E)*	*E*	*k(E)*	*E*	*k(E)*	*E*
i9 – p2	1.64E+04	230	3.95E+04	240	8.77E+04	250	1.81E+05	260	3.53E+05	270
i9 – p1	6.16E+03	230	1.81E+04	240	4.76E+04	250	1.14E+05	260	2.51E+05	270
i2 – p3	2.81E+05	254	5.73E+05	264	1.10E+06	274	2.02E+06	284	3.53E+06	294
i2 – p2	3.60E+04	254	8.36E+04	264	1.80E+05	274	3.66E+05	284	7.01E+05	294
i1 – i11	8.69E+01	255	2.07E+02	265	4.59E+02	275	9.61E+02	285	1.91E+03	295
i11 – i1	1.15E+01	263	2.77E+01	273	6.23E+01	283	1.32E+02	293	2.65E+02	303
i7 – i8	6.83E+06	338	9.58E+06	348	1.32E+07	358	1.79E+07	368	2.39E+07	378
i8 – i7	2.56E+12	210	2.72E+12	220	2.88E+12	230	3.04E+12	240	3.19E+12	250
i1 – i6	1.53E+03	255	3.73E+03	265	8.46E+03	275	1.80E+04	285	3.64E+04	295
i6 – i1	1.56E+01	298	4.16E+01	308	1.02E+02	318	2.36E+02	328	5.14E+02	338
i9 – i8	1.10E+08	230	1.53E+08	240	2.08E+08	250	2.77E+08	260	3.63E+08	270
i8 – i9	5.71E+09	210	7.51E+09	220	9.67E+09	230	1.22E+10	240	1.52E+10	250
i9 – i11	4.82E-01	230	1.91E+00	240	6.60E+00	250	2.02E+01	260	5.62E+01	270
i11 – i9	1.00E-02	263	4.28E-02	273	1.60E-01	283	5.27E-01	293	1.56E+00	303
i9 – i10	5.14E+00	230	2.03E+01	240	7.15E+01	250	2.26E+02	260	6.45E+02	270
i10 – i9	4.18E+00	219	1.61E+01	229	5.50E+01	239	1.70E+02	249	4.74E+02	259
i4 – p4	2.61E+06	242	5.24E+06	252	9.94E+06	262	1.79E+07	272	3.09E+07	282
i4 – i7	1.51E+04	242	3.09E+04	252	6.00E+04	262	1.11E+05	272	1.96E+05	282
i7 – i4	7.76E-01	338	1.92E+00	348	4.46E+00	358	9.79E+00	368	2.04E+01	378
i10 – p1	9.67E+02	219	2.98E+03	229	8.09E+03	239	1.99E+04	249	4.50E+04	259
i10 – p6	1.46E+08	219	2.34E+08	229	3.61E+08	239	5.38E+08	249	7.82E+08	259
i1 – i2	1.08E+12	255	1.16E+12	265	1.25E+12	275	1.33E+12	285	1.41E+12	295
i2 – i1	1.45E+12	254	1.55E+12	264	1.65E+12	274	1.75E+12	284	1.85E+12	294
i4 – i6	5.40E-07	242	2.99E-05	252	4.56E-04	262	3.85E-03	272	2.26E-02	282
i6 – i4	1.29E-09	298	8.04E-08	308	1.38E-06	318	1.30E-05	328	8.47E-05	338
i9 – i5	1.30E+03	230	3.11E+03	240	6.90E+03	250	1.43E+04	260	2.80E+04	270
i5 – i9	7.17E-03	365	2.22E-02	375	6.31E-02	385	1.66E-01	395	4.08E-01	405
i11 – p5	7.79E+06	263	1.40E+07	273	2.41E+07	283	4.00E+07	293	6.41E+07	303
i11 – p2	4.41E+03	263	1.08E+04	273	2.46E+04	283	5.23E+04	293	1.05E+05	303
i6 – p3	1.28E+04	298	2.61E+04	308	5.05E+04	318	9.34E+04	328	1.66E+05	338
i6 – p4	8.29E+05	298	1.59E+06	308	2.91E+06	318	5.11E+06	328	8.64E+06	338
i2 – i5	1.06E+03	254	2.51E+03	264	5.53E+03	274	1.14E+04	284	2.24E+04	294
i5 – i2	2.77E-02	365	8.03E-02	375	2.15E-01	385	5.33E-01	395	1.25E+00	405
i2 – i3	6.18E+07	254	8.85E+07	264	1.24E+08	274	1.69E+08	284	2.27E+08	294
i3 – i2	1.05E+11	182	1.22E+11	192	1.38E+11	202	1.56E+11	212	1.75E+11	222

i4 – i3	1.69E+08	242	2.35E+08	252	3.20E+08	262	4.26E+08	272	5.57E+08	282
i3 – i4	8.98E+10	182	1.04E+11	192	1.18E+11	202	1.33E+11	212	1.49E+11	222
i5 – p2	1.04E+00	365	2.96E+00	375	7.74E+00	385	1.88E+01	395	4.31E+01	405
i11 – i5	1.47E+00	263	4.31E+00	273	1.16E+01	283	2.92E+01	293	6.83E+01	303
i5 – i11	3.89E-04	365	1.37E-03	375	4.40E-03	385	1.30E-02	395	3.58E-02	405
i10 – i5	1.27E+04	219	2.53E+04	229	4.77E+04	239	8.56E+04	249	1.47E+05	259
i5 – i10	4.31E-02	365	1.14E-01	375	2.83E-01	385	6.62E-01	395	1.46E+00	405
i6 – i5	6.90E+02	298	1.44E+03	308	2.85E+03	318	5.39E+03	328	9.82E+03	338
i5 – i6	2.36E+00	365	5.48E+00	375	1.21E+01	385	2.53E+01	395	5.08E+01	405
i10 – i12	2.79E-01	219	1.00E+00	229	3.30E+00	239	9.91E+00	249	2.73E+01	259
i12 – i10	5.29E-02	244	2.02E-01	254	7.05E-01	264	2.23E+00	274	6.48E+00	284
i12 – p5	8.17E+07	244	1.34E+08	254	2.10E+08	264	3.21E+08	274	4.76E+08	284
i12 – p1	4.52E+02	244	1.33E+03	254	3.50E+03	264	8.40E+03	274	1.87E+04	284

Table S3. Statistical branching ratios (%) for all products of the reaction of phenylethynyl (C_6H_5CC) with allene (H_2CCCH_2) and with methylacetylene (H_3CCCH) at different collision energies (E_C, kJ mol^{-1}).

Phenylethynyl + Allene										
	Initial intermediate ***i7***					Initial intermediate ***i9***				
E E_C	**0**	**10**	**20**	**30**	**40**	**0**	**10**	**20**	**30**	**40**
p1	23.1	27.2	31.0	34.5	37.6	23.1	27.2	31.0	34.5	37.6
p2	57.4	56.5	55.1	53.5	51.9	57.4	56.5	55.1	53.5	51.9
p3	4.2	3.4	2.8	2.4	2.0	4.2	3.4	2.8	2.4	2.0
p4	15.2	12.8	11.0	9.5	8.4	15.2	12.8	11.0	9.5	8.3
p5	0.0	0.0	0.0	0.0	0.0	0.0	0.0	0.0	0.0	0.0
p6	0.1	0.1	0.1	0.1	0.2	0.1	0.1	0.1	0.1	0.2
Phenylethynyl + Methylacetylene										
	Initial intermediates ***i1/i2***					Initial intermediate ***i4***				
E_C	**0**	**10**	**20**	**30**	**40**	**0**	**10**	**20**	**30**	**40**
p1	0.1	0.1	0.1	0.1	0.2	0.1	0.1	0.1	0.2	0.2
p2	3.5	3.9	4.4	4.8	5.3	3.4	3.8	4.2	4.5	4.8
p3	25.3	25.1	25.0	25.0	25.0	24.6	24.1	23.6	23.1	22.7
p4	71.1	70.9	70.5	70.1	69.5	71.9	72.0	72.1	72.2	72.3
p5	0.0	0.0	0.0	0.0	0.0	0.0	0.0	0.0	0.0	0.0
p6	0.0	0.0	0.0	0.0	0.0	0.0	0.0	0.0	0.0	0.0

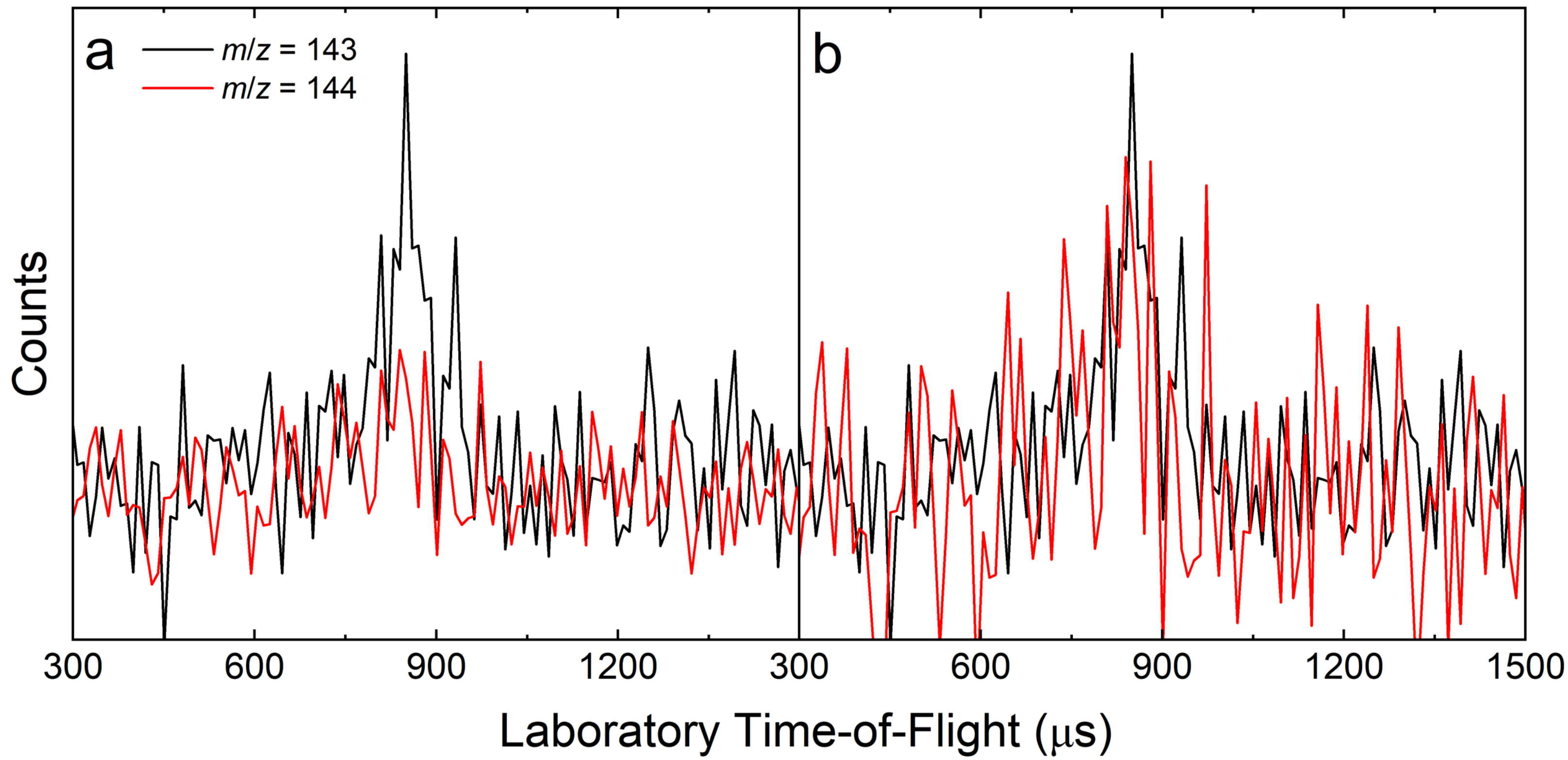

Figure S1. Time-of-flight (TOF) spectra for the reaction of phenylethynyl (C_6H_5CC) with allene-d_4 (D_2CCCD_2) taken at m/z = 143 (black) and 144 (red). Absolute counts are shown in (**a**) while the two spectra are scaled in (**b**).

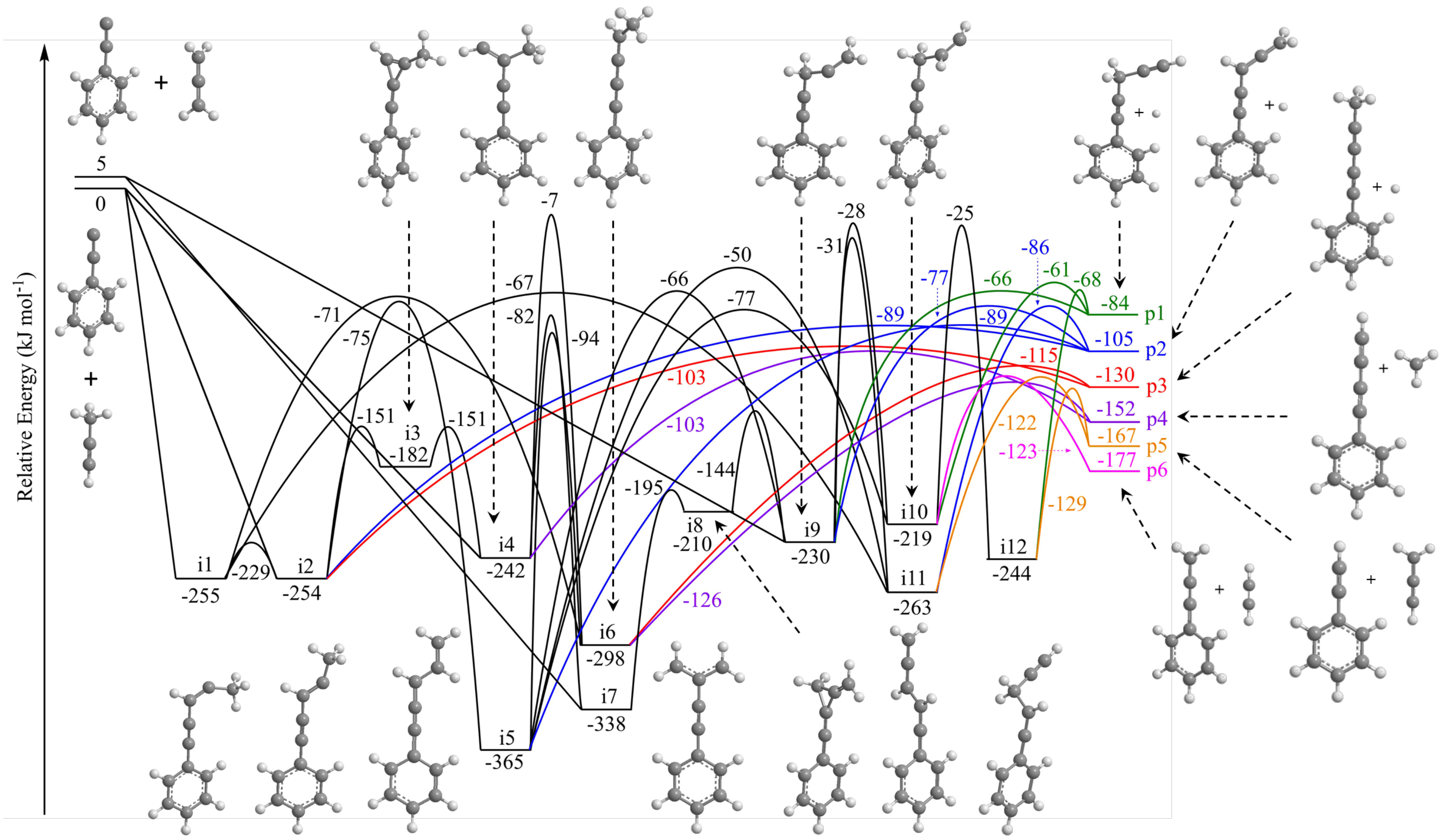

Figure S2. Calculated potential energy surface for the reaction of phenylethynyl (C_6H_5CC) with allene (H_2CCCH_2) and with methylacetylene (H_3CCCH) at the G3(MP2,CC)// ωB97X-D/6-311G(d,p) level. Energies are in units of kJ mol^{-1} and colored pathways denote exit channels to the products. Carbon atoms are gray and hydrogen atoms are white.

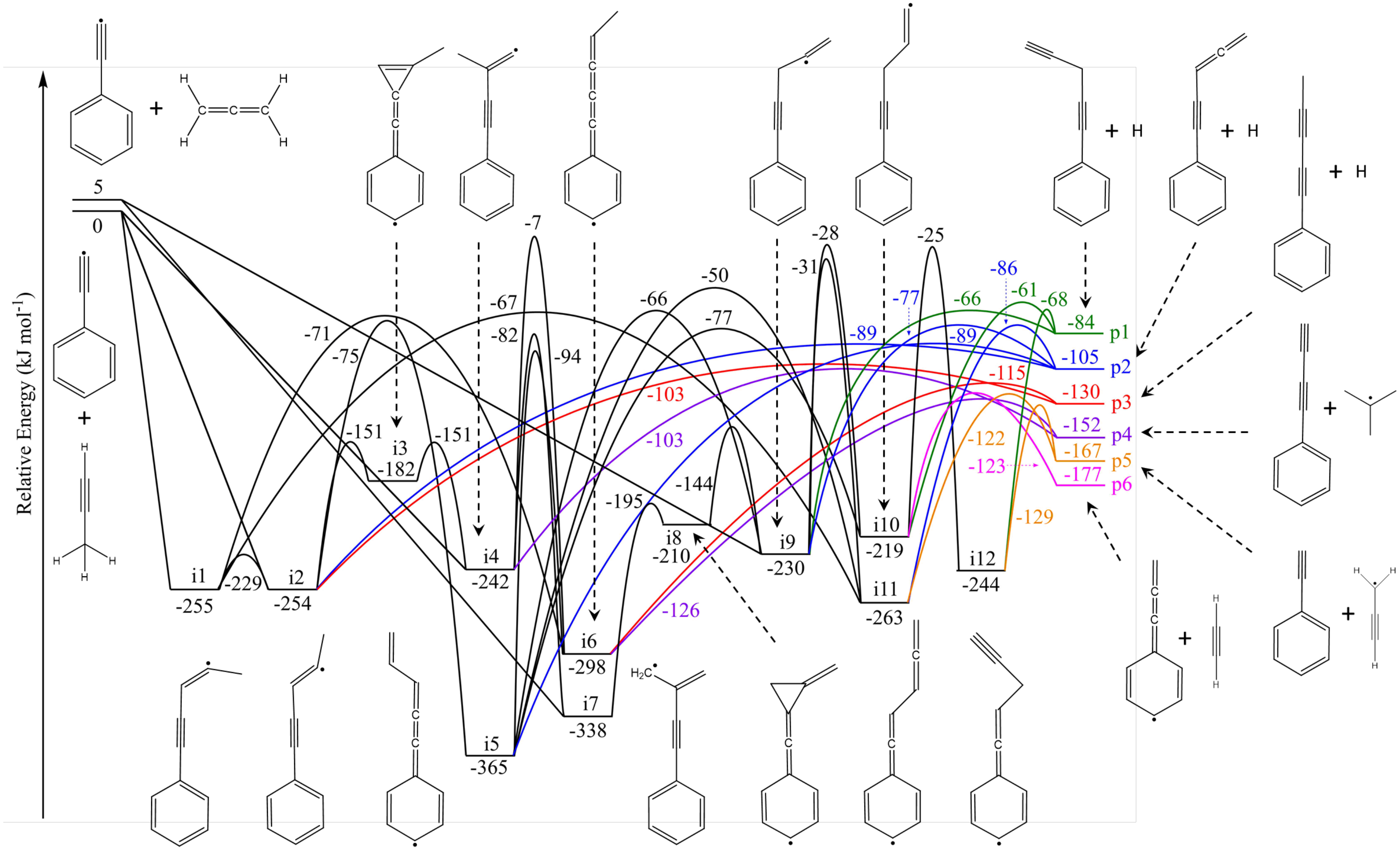

Figure S3. Calculated potential energy surface as shown in Figure S2 with two-dimensional structures.

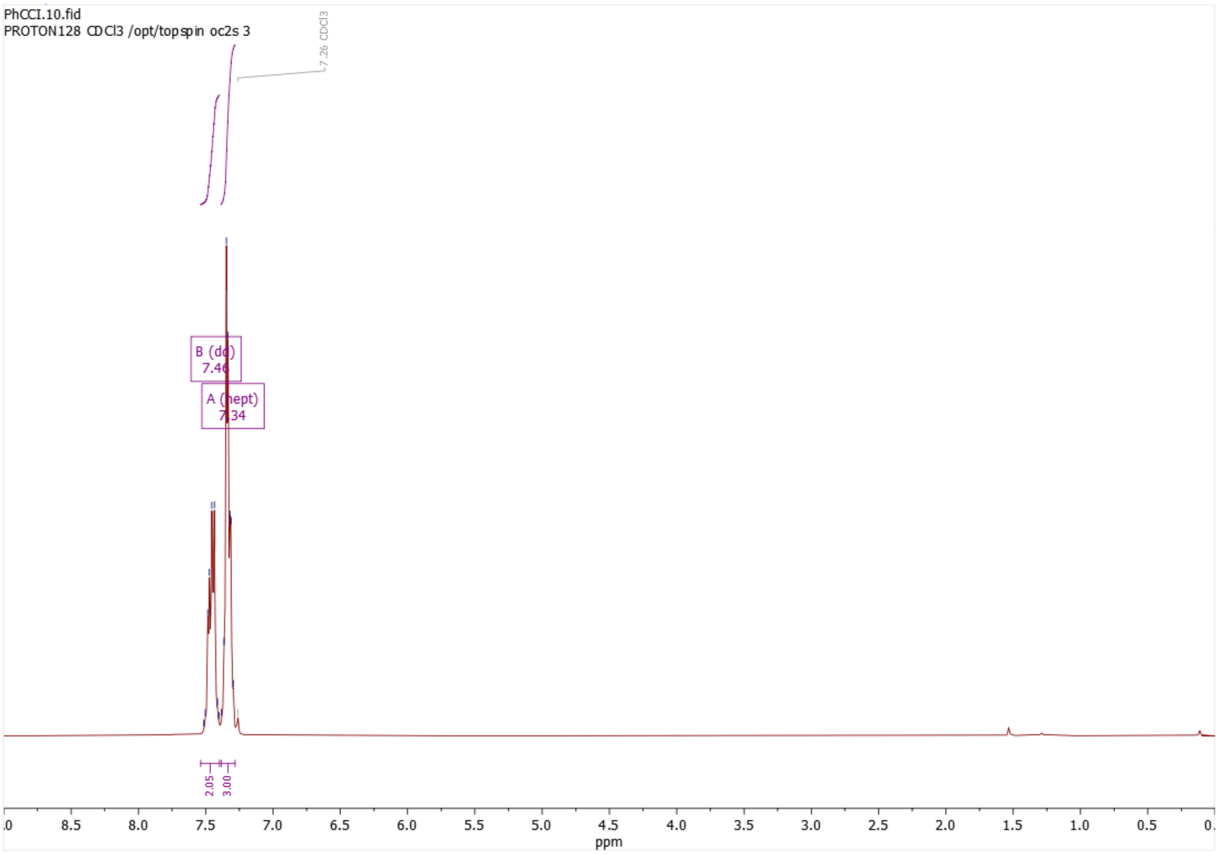

Figure S3. ^{1}H NMR spectrum of (2-iodoethynyl)benzene.

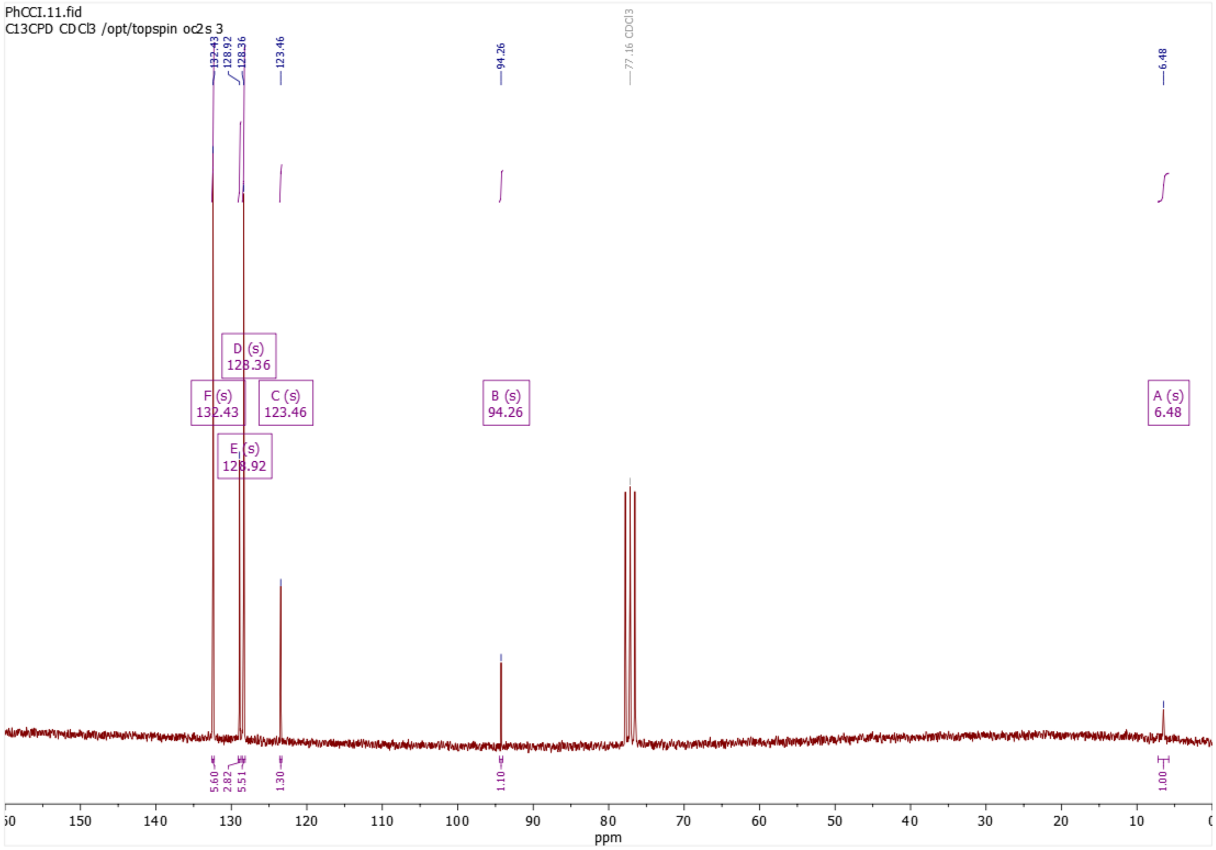

Figure S4. ^{13}C NMR spectrum of (2-iodoethynyl)benzene.

Optimized Cartesian coordinates (Å) and vibrational frequencies (cm^{-1}) for all intermediates, transition states, reactants, and products involved in the C_8H_5 + C_3H_4 reactions at the ωB97X-D/6-311G(d,p) level.

Reactants

C_8H_5

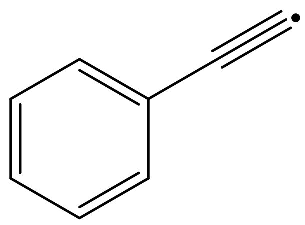

Cartesian coordinates

C	0.045775	1.219540	0.013382
C	1.426126	1.212650	-0.006714
C	2.115813	-0.000010	-0.017253
C	1.426105	-1.212662	-0.006715
C	0.045755	-1.219531	0.013384
C	-0.671544	0.000009	0.023596
C	-2.068345	0.000016	0.040729
C	-3.340546	-0.000008	-0.055969
H	-0.510694	2.148691	0.019942
H	1.973361	2.147570	-0.015716
H	3.199878	-0.000024	-0.035097
H	1.973339	-2.147581	-0.015717
H	-0.510719	-2.148680	0.019947

Frequencies

109.1212	143.9347	246.8164
390.7115	476.4153	484.9854
507.4479	629.6306	687.8436
783.4529	788.1472	862.7349

967.3232	1013.8868	1014.0002
1029.0644	1049.7885	1112.7315
1182.8967	1188.0328	1227.9584
1322.9787	1353.0819	1478.2436
1508.7173	1612.2257	1638.8824
1988.9682	3202.8066	3213.5662
3220.9948	3230.0795	3233.7590

Methylacetylene

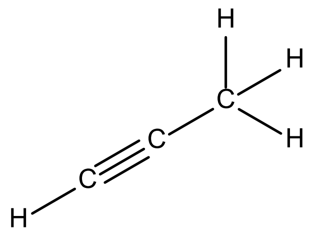

Cartesian coordinates

C	-0.219275	-0.000871	-0.000237
C	-1.417960	0.000091	0.000010
C	1.238342	0.000094	0.000115
H	-2.481089	0.001227	0.000307
H	1.624994	0.232644	-0.994855
H	1.626340	-0.976957	0.297065
H	1.623113	0.747205	0.698157

Frequencies

346.1945	348.5118	683.1649
683.3323	952.2647	1057.0201
1061.1503	1416.9837	1481.6045
1486.1575	2257.5004	3056.2365
3129.3053	3132.9810	3484.4471

Allene

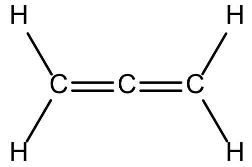

Cartesian coordinates

C	1.300828	0.000065	-0.000100
C	-0.000003	-0.000043	0.000099
C	-1.300848	0.000162	0.000179
H	1.860165	-0.617755	-0.694468
H	1.860571	0.617258	0.694514
H	-1.860086	0.694522	-0.617768
H	-1.860516	-0.695130	0.616650

Frequencies

374.2550	374.2736	885.8717
886.0208	892.1538	1020.4348
1020.4726	1117.3684	1422.7168
1485.3699	2081.8102	3152.6728
3154.9596	3237.1291	3237.1866

Intermediates

i1

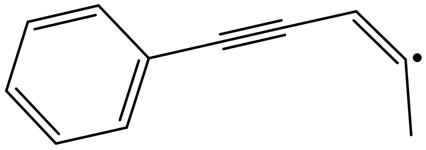

Cartesian coordinates

C	-1.866871	1.217741	0.066268
C	-3.217169	0.902718	0.085219
C	-3.628453	-0.424034	0.022609

C	-2.680721	-1.437726	-0.059520
C	-1.328644	-1.130446	-0.078946
C	-0.906321	0.202815	-0.016463
C	0.486027	0.522043	-0.038216
C	1.661342	0.792252	-0.059621
C	3.996015	0.181557	-0.010107
C	3.055302	1.104415	-0.088160
C	4.104198	-1.274978	0.120975
H	-1.542000	2.250209	0.116031
H	-3.952763	1.696376	0.148994
H	-4.684887	-0.666345	0.037652
H	-2.996206	-2.473662	-0.109949
H	-0.586672	-1.917403	-0.143696
H	3.325519	2.154157	-0.181575
H	4.606100	-1.710659	-0.747498
H	3.104741	-1.723616	0.204467
H	4.677939	-1.547197	1.011348

Frequencies

20.5526	58.6745	86.0164
118.0515	128.4693	233.9918
310.5158	360.9435	381.0915
413.9244	450.7137	537.4562
556.4986	625.8414	644.7342
711.0825	745.2729	780.9028
809.8854	866.5720	911.7574
943.3511	1000.3561	1017.8434
1021.1900	1024.8066	1045.1794
1061.0372	1068.8140	1112.9055
1188.7191	1208.7490	1285.1840

1317.7799	1321.8541	1356.2133
1399.0735	1461.5067	1470.9684
1488.5923	1542.4382	1647.0311
1678.8879	1737.0315	2346.5316
3008.2324	3091.2696	3116.3588
3152.5443	3191.0583	3200.9220
3209.9129	3218.1493	3223.4995

i2

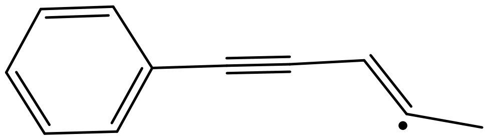

Cartesian coordinates

C	1.943497	-1.203388	0.014566
C	3.318386	-1.023825	0.021706
C	3.857948	0.257508	0.010333
C	3.013957	1.362368	-0.007683
C	1.637941	1.190784	-0.014676
C	1.086991	-0.096144	-0.003797
C	-0.329897	-0.277415	-0.013114
C	-1.526790	-0.419822	-0.022468
C	-2.934965	-0.631843	-0.040264
H	1.518024	-2.199816	0.023594
H	3.972729	-1.888097	0.035958
H	4.933175	0.395159	0.015777
H	3.430606	2.363139	-0.016933
H	0.975356	2.047981	-0.028946
H	-3.267917	-1.672355	-0.118223
C	-3.842866	0.321866	0.026918
C	-5.304135	0.442392	0.028889

H	-5.659420	0.904774	0.954167
H	-5.775186	-0.546730	-0.062802
H	-5.647754	1.061058	-0.805057

Frequencies

31.9807	59.7647	76.6186
148.0435	154.4828	191.0489
284.8947	295.6726	363.4684
413.4081	433.8967	523.5969
555.1421	584.1764	643.7912
710.3673	747.7393	779.4855
796.5435	866.8113	920.7189
943.2506	1001.4078	1017.0283
1023.9584	1045.0231	1047.2666
1065.1884	1073.3376	1112.2766
1188.9262	1206.2322	1266.5415
1316.5845	1325.6720	1355.4267
1403.7355	1457.0294	1477.6827
1488.9912	1541.0459	1647.0062
1678.7228	1755.0991	2355.0228
3000.8700	3063.4442	3088.8445
3115.0075	3191.7889	3201.4334
3211.6677	3220.6157	3225.4730

i3

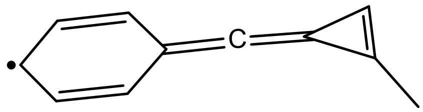

Cartesian coordinates

C	1.526427	-1.216317	-0.051405
C	2.891238	-1.205832	0.153445
C	3.586810	0.000119	0.258574
C	2.891052	1.205964	0.153457
C	1.526239	1.216236	-0.051387
C	0.793599	-0.000097	-0.159111
C	-0.571672	-0.000175	-0.362211
C	-1.846751	-0.000065	-0.496020
H	0.988988	-2.153881	-0.133909
H	3.426201	-2.146026	0.233997
H	4.658518	0.000202	0.419956
H	3.425867	2.146242	0.234015
H	0.988651	2.153716	-0.133882
C	-3.209995	0.000017	0.039030
C	-3.088140	0.000099	-1.264395
C	-4.036881	-0.000007	1.258442
H	-3.554819	0.000235	-2.236482
H	-3.801013	0.879547	1.863109
H	-3.802220	-0.880511	1.862202
H	-5.101724	0.000828	1.020470

Frequencies

38.1395	41.1010	57.5982
103.6750	168.2127	246.7127
261.6050	371.6465	416.7166
435.6177	472.4259	493.8407

496.0675	631.2155	679.6607
694.7710	719.7154	759.1626
764.7686	839.1064	910.3111
911.2774	984.7165	988.2985
1000.2295	1003.5586	1041.7085
1048.0781	1053.0723	1106.7622
1145.4102	1175.5430	1197.1733
1300.0843	1317.5915	1347.8369
1407.2294	1472.1032	1474.5932
1478.5049	1505.9901	1592.8436
1619.4679	1791.3992	1988.1866
3051.0278	3122.1431	3144.6560
3187.1551	3193.3643	3208.9722
3213.6255	3221.1225	3278.1397

i4

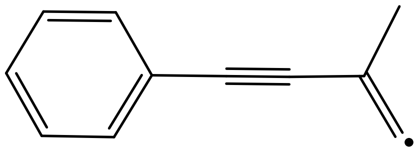

Cartesian coordinates

C	1.527460	-1.197082	-0.066832
C	2.913083	-1.249419	-0.066024
C	3.657918	-0.077346	0.001435
C	3.010243	1.150836	0.068990
C	1.624694	1.211360	0.068083
C	0.868202	0.035722	0.000052
C	-0.559704	0.090535	-0.003229
C	-1.764807	0.135848	-0.008673
C	-3.198463	0.157744	-0.014908
C	-3.834873	1.307471	-0.131218

C	-3.909563	-1.169763	0.115998
H	0.943048	-2.107925	-0.119819
H	3.413854	-2.209252	-0.119672
H	4.741026	-0.121600	0.001742
H	3.586490	2.067446	0.122351
H	1.115659	2.166286	0.121138
H	-3.564692	2.348709	-0.236111
H	-3.608044	-1.839493	-0.693532
H	-4.990406	-1.031699	0.080007
H	-3.642081	-1.647903	1.061858

Frequencies

21.7254	62.4932	68.4786
149.9013	171.1113	216.8517
281.2870	375.7872	413.1299
453.5372	482.7813	519.4341
564.1217	585.6346	642.7695
698.0218	710.9193	710.9905
781.4076	828.3125	867.1153
909.2449	945.6057	1001.4021
1018.7996	1023.7609	1037.2860
1057.1279	1062.5678	1112.3537
1178.4095	1188.0368	1212.2778
1318.6382	1328.2735	1356.9085
1412.6475	1480.0955	1487.8833
1488.6311	1543.2989	1646.5775
1667.7859	1681.9136	2346.4793
3056.9169	3128.3822	3156.9245
3193.8456	3202.1910	3212.3174
3219.7659	3225.5886	3262.1045

i5

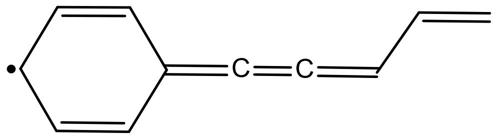

Cartesian coordinates

C	-1.896015	-1.216595	-0.000016
C	-3.270371	-1.042727	-0.000014
C	-3.816173	0.237003	0.000002
C	-2.977749	1.347338	0.000015
C	-1.601980	1.184047	0.000011
C	-1.041934	-0.102784	-0.000004
C	0.366035	-0.276891	-0.000002
C	1.575126	-0.428890	0.000010
C	2.946396	-0.607958	0.000027
C	3.875160	0.461098	-0.000033
C	5.220362	0.292661	0.000001
H	-1.465431	-2.210762	-0.000029
H	-3.921080	-1.909763	-0.000025
H	-4.892006	0.369192	0.000005
H	-3.400319	2.345571	0.000029
H	-0.944337	2.045137	0.000023
H	3.325618	-1.626455	0.000096
H	3.466303	1.468001	-0.000112
H	5.895082	1.139489	-0.000047
H	5.663030	-0.698220	0.000082

Frequencies

30.7495	55.6762	59.9992
142.6306	165.3310	285.9414

321.7522	409.7708	412.4060
440.0543	529.9652	542.0810
595.5511	597.8958	642.5782
706.0432	758.4969	768.2109
776.4393	863.2020	877.4851
939.8836	946.3990	1000.4697
1006.2164	1016.6905	1020.0918
1055.7005	1082.1061	1112.9420
1187.7332	1188.5878	1205.8293
1281.6824	1301.8553	1315.8315
1354.9155	1387.0728	1465.3605
1487.3596	1541.7654	1560.8710
1638.8649	1667.5470	2172.3185
3160.4151	3172.9310	3183.8370
3192.7671	3202.0582	3211.9128
3221.7102	3226.4493	3259.3604

i6

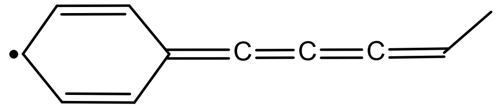

Cartesian coordinates

C	-1.801597	-1.212323	-0.013201
C	-3.180585	-1.204232	0.104476
C	-3.876344	0.000184	0.164374
C	-3.180352	1.204456	0.104304
C	-1.801363	1.212257	-0.013378
C	-1.086670	-0.000107	-0.073078
C	0.314242	-0.000217	-0.188207
C	1.548805	-0.000221	-0.282785

C	4.157909	-0.000075	-0.465574
C	2.854414	-0.000178	-0.386936
C	5.094014	0.000245	0.714206
H	-1.257878	-2.148200	-0.059994
H	-3.718385	-2.144344	0.150803
H	-4.956040	0.000294	0.258663
H	-3.717962	2.144682	0.150509
H	-1.257455	2.148018	-0.060297
H	4.622547	-0.000182	-1.453187
H	5.742538	-0.880923	0.686019
H	4.545603	0.000272	1.656509
H	5.742201	0.881656	0.685776

Frequencies

38.6312	45.9070	88.7615
96.8998	116.9881	221.4186
244.9759	338.6318	404.6867
411.4858	475.7924	483.6153
519.3581	570.9122	637.9239
695.4548	704.4176	724.8039
774.1558	855.2508	877.4750
934.9643	996.0218	1010.8892
1015.7718	1040.0572	1051.4500
1059.8388	1098.7364	1112.0776
1184.5588	1207.3999	1265.8438
1311.8084	1355.7783	1384.6957
1405.7216	1481.9200	1485.9817
1490.3463	1527.3906	1624.4442
1646.4175	1907.4788	2054.1240
3040.9401	3102.7663	3111.3226

3152.6080	3194.4934	3203.0751
3213.6132	3221.0572	3226.2850

i7

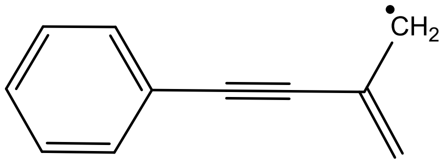

Cartesian coordinates

C	1.586899	1.205971	0.046927
C	2.973717	1.202017	0.047041
C	3.670537	-0.000001	0.000002
C	2.973716	-1.202018	-0.047039
C	1.586898	-1.205971	-0.046929
C	0.878418	0.000001	-0.000002
C	-0.551111	0.000001	-0.000003
C	-1.755644	0.000000	-0.000003
C	-3.862130	1.215052	-0.083798
C	-3.193214	0.000000	0.000000
C	-3.862129	-1.215053	0.083802
H	1.039857	2.140485	0.083861
H	3.512540	2.141867	0.084364
H	4.754579	-0.000001	0.000004
H	3.512538	-2.141868	-0.084360
H	1.039855	-2.140483	-0.083864
H	-3.322237	2.149957	-0.150099
H	-4.945320	1.241920	-0.084851
H	-3.322234	-2.149958	0.150099
H	-4.945318	-1.241921	0.084862

Frequencies

15.8263	56.0266	66.6903
177.9849	205.8122	297.3887
378.1348	413.2230	470.9354
500.3772	524.6132	531.9952
546.5506	601.8323	625.9806
643.0375	698.0043	710.7544
761.9773	781.1118	789.9572
866.3098	912.6641	945.3882
981.6417	1000.5831	1018.0771
1022.7152	1042.9662	1062.0190
1111.7196	1187.4483	1211.6021
1252.9520	1288.8184	1318.7223
1356.7087	1411.5763	1475.5573
1487.2479	1527.1680	1548.9876
1646.3827	1679.1422	2359.9001
3168.5147	3174.4619	3195.1878
3203.5742	3213.8008	3220.6425
3226.7153	3278.5467	3280.1039

i8

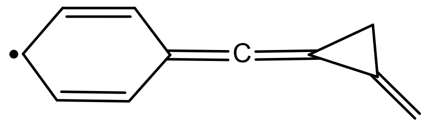

Cartesian coordinates

C	-1.497681	1.219995	-0.008560
C	-2.872733	1.206733	0.100850
C	-3.572928	0.000234	0.157152
C	-2.873052	-1.206463	0.101164
C	-1.498000	-1.220125	-0.008239

C	-0.767170	-0.000170	-0.066185
C	0.604910	-0.000299	-0.166740
C	1.876756	-0.000252	-0.312635
C	3.212976	0.000038	0.201214
C	3.061643	-0.000171	-1.273140
C	4.017493	0.000319	1.246442
H	-0.955832	2.157182	-0.051379
H	-3.412103	2.146425	0.144008
H	-4.652829	0.000389	0.245518
H	-3.412679	-2.145999	0.144552
H	-0.956410	-2.157472	-0.050826
H	3.261801	-0.919478	-1.817766
H	3.261447	0.919056	-1.818030
H	3.618590	0.000325	2.254331
H	5.094742	0.000536	1.121654

Frequencies

31.0185	58.1739	82.8647
174.9840	235.8392	260.4182
362.0532	364.0447	411.8284
457.5039	487.2522	540.1295
631.7287	668.8393	675.0716
689.2627	737.7980	757.7078
834.1880	897.4042	904.7317
917.7164	945.9584	960.0238
987.6087	1002.2174	1003.3324
1035.4549	1047.4941	1048.0417
1110.7024	1133.8119	1178.0116
1197.2857	1294.9681	1303.6357
1350.6928	1432.3372	1464.2404

1471.4423	1504.4960	1593.4801
1619.3877	1809.0019	1975.0650
3109.3931	3158.1577	3192.6874
3199.4326	3199.8194	3213.0089
3218.8448	3224.9283	3251.4445

i9

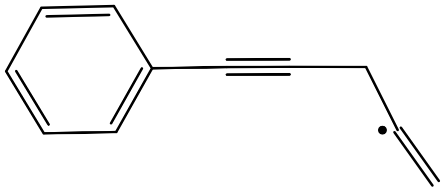

Cartesian coordinates

C	1.916371	-1.196651	0.072543
C	3.273294	-0.912238	0.116639
C	3.715226	0.404321	0.053040
C	2.792628	1.438487	-0.055547
C	1.434554	1.161300	-0.101110
C	0.982429	-0.161204	-0.037228
C	-0.420290	-0.444821	-0.083535
C	-1.600490	-0.667489	-0.124324
C	-3.038677	-0.931866	-0.175570
C	-3.852398	0.142054	0.441145
C	-4.751451	1.010006	0.061168
H	1.566997	-2.221104	0.123722
H	3.989605	-1.721482	0.201296
H	4.776312	0.623097	0.089159
H	3.132662	2.466627	-0.105752
H	0.710377	1.962836	-0.184707
H	-3.248240	-1.885062	0.323021
H	-3.349872	-1.056067	-1.224989

H	-5.213654	1.704091	0.757234
H	-5.071358	1.075671	-0.982314

Frequencies

22.0444	42.0554	75.5169
98.8458	169.1591	259.9868
308.1030	358.1712	399.5048
413.9447	434.6829	545.6109
555.3945	606.1041	642.9403
712.2996	742.1912	782.2437
867.7284	871.3510	900.9986
925.4954	946.2860	1001.2710
1007.8859	1018.5980	1024.2334
1055.0556	1064.3031	1111.3262
1188.7974	1207.0222	1220.7231
1283.7245	1316.8106	1333.1355
1355.2038	1413.5742	1452.8987
1488.0070	1541.4943	1648.1465
1680.1384	1763.2156	2386.0095
2988.8551	3066.3774	3073.1366
3186.3459	3192.4445	3201.2446
3211.4112	3218.6062	3224.2903

i10

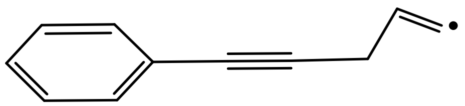

Cartesian coordinates

C	1.754546	-1.195290	0.113432
C	3.121786	-1.056095	0.300438

C	3.712814	0.200427	0.235421
C	2.929492	1.320747	-0.017846
C	1.561553	1.188605	-0.205303
C	0.959465	-0.072591	-0.141576
C	-0.452385	-0.213440	-0.333809
C	-1.637753	-0.331775	-0.499453
C	-3.081012	-0.475275	-0.671145
C	-3.846767	-0.133913	0.602662
C	-4.784237	0.770351	0.677751
H	1.288908	-2.172319	0.163737
H	3.728171	-1.932697	0.498042
H	4.781403	0.307313	0.382577
H	3.386527	2.302312	-0.069648
H	0.946650	2.058838	-0.401715
H	-3.305954	-1.510707	-0.950579
H	-3.422565	0.159940	-1.492908
H	-3.560921	-0.704325	1.485857
H	-5.267233	1.481133	0.021196

Frequencies

6.1724	53.2133	79.5552
106.6822	165.3351	303.0153
322.3224	351.7360	414.6719
421.4374	502.0102	548.2856
569.8212	642.0793	678.9391
716.3134	744.3362	788.5389
826.4770	862.6284	867.0886
946.4170	952.8848	1000.8200
1004.9219	1022.9500	1026.6297
1036.9391	1063.8338	1112.6047

1189.7605	1208.7334	1215.5677
1274.8104	1290.0201	1317.6307
1338.5649	1356.4033	1463.7471
1489.3574	1543.1820	1648.2166
1680.5453	1691.9805	2380.2837
3048.1113	3100.4450	3134.0289
3193.0557	3202.2712	3212.1402
3219.9046	3225.2378	3252.7345

i11

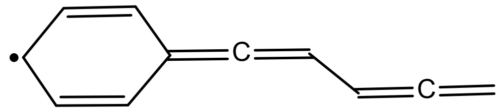

Cartesian coordinates

C	1.695516	1.222131	-0.086880
C	3.058472	1.207906	0.119060
C	3.753215	0.000431	0.224799
C	3.059038	-1.207331	0.119340
C	1.696135	-1.222161	-0.086569
C	0.967217	-0.000259	-0.195648
C	-0.387493	-0.000494	-0.395419
C	-1.677120	-0.000722	-0.585616
C	-3.966395	0.000063	0.283143
C	-2.676276	-0.000372	0.498071
C	-5.246899	0.000613	0.057208
H	1.157957	2.159184	-0.169189
H	3.594148	2.147202	0.200298
H	4.824453	0.000717	0.387550
H	3.595033	-2.146432	0.200740
H	1.158992	-2.159465	-0.168692

H	-2.308077	0.000047	1.520217
H	-5.800008	-0.928739	-0.038235
H	-2.075906	-0.001723	-1.602162
H	-5.799059	0.930377	-0.039466

Frequencies

18.6480	36.6877	104.7945
156.1217	185.5417	187.6805
340.9825	341.7247	410.8852
469.1215	494.2605	532.0186
580.9634	593.1928	629.7887
688.9884	761.0925	795.1152
831.7858	849.0917	896.0317
907.8826	909.6704	987.4334
989.8843	1005.7508	1019.1038
1038.4811	1045.7004	1110.0051
1133.5291	1167.0110	1176.4957
1214.5926	1263.0657	1301.4224
1349.6575	1389.9093	1468.7482
1482.1239	1501.6521	1587.8705
1614.6482	1895.7713	2071.8308
3103.3084	3142.7649	3177.3472
3192.9566	3199.1970	3213.5649
3218.6648	3224.9295	3225.1946

i12

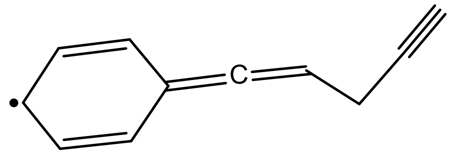

Cartesian coordinates

C	-1.804639	-1.207999	0.003432
C	-3.127240	-0.906611	0.249077
C	-3.566701	0.419507	0.267212
C	-2.655268	1.452436	0.034306
C	-1.327384	1.178993	-0.213707
C	-0.858942	-0.168081	-0.235771
C	0.458884	-0.448065	-0.489635
C	1.720645	-0.691365	-0.660625
C	2.738042	-0.772288	0.470053
C	3.816616	0.200023	0.301444
C	4.692557	1.002765	0.141317
H	-1.464967	-2.236813	-0.012190
H	-3.833094	-1.709761	0.429389
H	-4.608778	0.644663	0.459887
H	-2.993591	2.482587	0.048088
H	-0.619702	1.979173	-0.394492
H	2.123582	-0.844986	-1.663369
H	3.169415	-1.779044	0.495712
H	2.239753	-0.609088	1.428606
H	5.467968	1.717385	0.005743

Frequencies

22.2220	44.1733	74.0020
132.9458	185.8542	244.7528
344.3546	364.4510	410.6418
468.2702	498.8388	540.7598

550.3158	633.1193	687.8849
706.1736	715.4441	751.0485
759.3165	813.3357	830.5268
905.4687	939.1431	964.1518
984.0921	989.8908	1002.0263
1029.3173	1044.0360	1110.5942
1157.3696	1178.2758	1208.1202
1225.4937	1303.0284	1307.1316
1348.5568	1368.9347	1468.5153
1471.7807	1495.4498	1591.3770
1614.4843	1920.5525	2252.3828
3056.2008	3099.4444	3113.7916
3190.5790	3197.0685	3210.3846
3216.3414	3222.5481	3485.3129

Transition states

i9 – p2

Cartesian coordinates

C	-1.908614	-1.191460	-0.089600
C	-3.270724	-0.931876	-0.095529
C	-3.735009	0.376071	-0.013031
C	-2.829344	1.427161	0.075280
C	-1.465741	1.175925	0.081427
C	-0.991346	-0.138280	-0.000839
C	0.413552	-0.399024	0.007512
C	1.599156	-0.606439	0.020247
C	3.002715	-0.881821	0.021801
C	3.915117	0.076048	-0.119240
C	4.828396	0.994345	-0.124113
H	-1.541450	-2.208865	-0.153520

H	-3.974062	-1.753894	-0.163649
H	-4.800524	0.575214	-0.017310
H	-3.187494	2.448191	0.141120
H	-0.754832	1.990605	0.150234
H	3.307023	-1.910814	-0.152380
H	3.154717	-1.333641	1.853163
H	5.171062	1.452559	-1.047238
H	5.276610	1.336745	0.806094

Frequencies

-917.4723	26.8895	47.6026
84.2680	135.9517	209.1841
252.2584	328.7978	358.3735
373.2181	413.3896	442.9981
481.1165	543.5969	549.6081
609.3710	632.8057	643.8005
710.8056	760.0490	780.3710
868.5764	899.4346	944.9632
956.0667	982.3768	994.3922
1002.4793	1018.5715	1023.4059
1059.8276	1108.4831	1111.4600
1189.2408	1206.2160	1282.5496
1316.6119	1354.9035	1366.8557
1460.8764	1487.7641	1541.5630
1646.9372	1678.6475	2009.0563
2365.2743	3126.2013	3164.4318
3194.1532	3203.1473	3208.6515
3212.9749	3220.4482	3225.9085

i9 – p1

Cartesian coordinates

C	-1.930684	-1.196688	-0.071716
C	-3.283213	-0.890584	-0.104902
C	-3.702832	0.433589	-0.049032
C	-2.762959	1.453901	0.040653
C	-1.409059	1.155525	0.074438
C	-0.980180	-0.174880	0.018590
C	0.418378	-0.479930	0.053730
C	1.595250	-0.717986	0.084150
C	3.029003	-1.010386	0.124196
C	3.861630	0.191718	0.097626
C	4.616593	1.124600	-0.034711
H	-1.598804	-2.227227	-0.116190
H	-4.013252	-1.688786	-0.174616
H	-4.760569	0.669132	-0.075967
H	-3.085734	2.487791	0.084922
H	-0.671435	1.946047	0.143256
H	3.300069	-1.643916	-0.729390
H	3.261065	-1.587932	1.025813
H	5.699895	0.674162	-1.613794
H	5.157202	2.037453	0.057846

Frequencies

-747.8847	16.2105	51.8250
71.0573	88.3716	138.1942
235.2363	239.5011	323.0405
367.5373	395.4066	413.6091
444.3888	539.4065	555.6114
605.3536	643.8467	699.5281

712.0260	743.5876	781.7729
811.8756	867.9212	922.4427
936.9938	946.5204	1001.8861
1019.2746	1021.7762	1024.7884
1064.4823	1112.7810	1189.2465
1208.7179	1245.8551	1289.3512
1318.6528	1356.4048	1359.6274
1450.9121	1488.9656	1543.2500
1649.0541	1681.0086	2188.0742
2390.2343	3036.8345	3075.2187
3192.1602	3201.5941	3210.8078
3218.8901	3224.1523	3457.1955

i2 – p3

Cartesian coordinates

C	-1.894481	-1.199522	0.011588
C	-3.279213	-1.126831	0.014803
C	-3.915605	0.109206	0.005456
C	-3.161580	1.277343	-0.006846
C	-1.776524	1.213819	-0.009894
C	-1.129568	-0.027257	-0.000746
C	0.296209	-0.096420	-0.004819
C	1.501306	-0.147919	-0.008429
C	4.054368	0.108527	0.012113
C	2.878724	-0.214736	-0.015288
C	5.497721	0.251892	0.019594
H	-1.392394	-2.159473	0.018641
H	-3.863783	-2.039589	0.024425
H	-4.998298	0.162766	0.007869
H	-3.654815	2.242455	-0.014341

H	-1.183503	2.120500	-0.019506
H	3.019619	-2.071027	-0.184119
H	5.843016	0.680658	0.963576
H	5.829038	0.901630	-0.794248
H	5.972973	-0.726528	-0.107492

Frequencies

-909.3028	35.3723	57.7765
67.0984	110.8614	149.4608
156.9670	277.0852	285.4684
332.7100	396.2346	412.3413
482.6347	512.5206	546.1420
572.9173	584.2069	642.4461
672.9299	710.2316	782.2650
845.5520	867.2942	948.3495
1002.3978	1019.8627	1023.9706
1042.3051	1055.3853	1060.8945
1113.0294	1137.5128	1188.8031
1211.9278	1319.0178	1343.5498
1357.6545	1418.7884	1474.2477
1479.3273	1488.2597	1544.5097
1646.5537	1679.5672	2223.0143
2379.4960	3038.4626	3109.8698
3126.4906	3193.8870	3202.9917
3211.7682	3222.6561	3227.3741

i2 – p2

Cartesian coordinates

C	1.959160	-1.198959	0.000591
C	3.322802	-0.947391	0.000864

C	3.793736	0.360768	0.000367
C	2.893291	1.420102	-0.000399
C	1.528069	1.176837	-0.000671
C	1.046737	-0.137542	-0.000183
C	-0.358961	-0.391950	-0.000488
C	-1.546444	-0.595337	-0.000759
C	-3.873729	0.045724	0.000134
C	-2.942601	-0.874376	-0.001162
C	-4.872823	0.894046	0.001371
H	1.587466	-2.216658	0.000982
H	4.021285	-1.776294	0.001468
H	4.860254	0.554283	0.000575
H	3.256161	2.441526	-0.000794
H	0.822097	1.998670	-0.001273
H	-3.246442	-1.920018	-0.002655
H	-6.533695	-0.245981	-0.000315
H	-5.231275	1.327837	-0.926731
H	-5.231268	1.325103	0.930750

Frequencies

-689.5498	22.5744	48.2074
85.3278	131.5218	175.6017
255.6387	279.7593	352.1639
367.4631	405.9602	414.3967
422.4994	544.8128	555.5986
623.4673	643.8126	692.6658
714.5254	758.2076	785.7166
869.9520	900.3443	934.5146
950.8103	981.4256	1003.8532
1017.5847	1024.0925	1024.1343

1061.4469	1112.5523	1118.7077
1189.2742	1209.4344	1288.0442
1319.2935	1356.4533	1372.6433
1469.5480	1488.5812	1544.3384
1647.5466	1680.4523	2024.8995
2361.3066	3137.3645	3140.6447
3188.8424	3197.4583	3207.7718
3214.7982	3220.4648	3225.4648

i1 – i11

Cartesian coordinates

C	1.573289	-1.212312	-0.038017
C	2.949133	-1.205685	0.112448
C	3.641400	0.000008	0.188143
C	2.949130	1.205695	0.112396
C	1.573286	1.212312	-0.038071
C	0.861385	-0.000003	-0.114328
C	-0.537713	-0.000011	-0.271715
C	-1.786310	-0.000014	-0.215907
C	-4.098275	-0.000007	-0.261668
C	-3.031926	-0.000052	-1.035643
C	-4.040223	0.000052	1.127398
H	1.027830	-2.146421	-0.097843
H	3.487535	-2.144543	0.171608
H	4.718876	0.000012	0.305607
H	3.487531	2.144557	0.171516
H	1.027826	2.146418	-0.097939
H	-2.977726	-0.000105	-2.115976
H	-4.289662	0.914271	1.665309
H	-2.511592	0.000040	0.942123

H	-4.289664	-0.914125	1.665381

Frequencies

-2223.1903	47.1183	59.4539
80.8943	201.6975	217.7650
315.0989	337.2880	394.2870
413.3474	516.6704	517.5798
565.8576	636.8506	667.2004
676.7047	705.4269	780.8768
827.2572	860.3101	864.0961
945.4649	948.2098	1003.1184
1003.6712	1014.4417	1018.6197
1022.3469	1057.8040	1101.4456
1105.0868	1112.7297	1187.7652
1202.9496	1244.6282	1282.9749
1316.2079	1354.6527	1470.8612
1483.8852	1523.4552	1533.2703
1630.3634	1656.5901	1735.6269
2050.1709	3104.0259	3180.4766
3190.1447	3198.8708	3209.0031
3216.4579	3221.5559	3233.4499

i7 – i8

Cartesian coordinates

C	1.520186	-1.212398	0.068856
C	2.903177	-1.203901	0.024595
C	3.602858	-0.000004	0.001973
C	2.903187	1.203899	0.024515
C	1.520196	1.212409	0.068774
C	0.801112	0.000009	0.089767

C	-0.601697	0.000015	0.139612
C	-1.839113	0.000007	0.029221
C	-3.248994	0.000001	0.083679
C	-3.042726	-0.000087	-1.363201
C	-4.215216	0.000062	0.988680
H	0.975283	-2.148655	0.087301
H	3.442663	-2.144180	0.007263
H	4.685997	-0.000010	-0.032112
H	3.442681	2.144173	0.007125
H	0.975301	2.148672	0.087161
H	-3.049671	0.933191	-1.912223
H	-3.049642	-0.933437	-1.912103
H	-3.985955	0.000135	2.047137
H	-5.254478	0.000041	0.681628

Frequencies

-664.6794	50.4206	54.5332
70.4485	186.2794	228.0607
280.8288	302.9319	350.6726
412.2697	457.4451	508.6794
535.7559	616.7028	640.1096
703.9995	704.6498	741.7038
761.0624	770.4324	855.8471
927.6165	934.1152	936.2755
959.4033	996.0669	1013.4116
1014.0388	1025.0921	1056.7898
1112.5293	1171.8175	1184.7700
1205.2864	1312.1269	1312.3983
1354.6215	1419.1605	1456.2670
1482.0901	1525.3010	1623.7789

1647.3600	1781.3986	2061.4842
3152.1320	3163.2985	3187.6856
3195.2190	3207.3159	3213.5497
3219.5920	3261.8223	3266.1141

i1 – i6

Cartesian coordinates

C	1.804139	-1.208300	0.005922
C	3.184378	-1.202871	-0.121985
C	3.879165	0.000131	-0.186214
C	3.184274	1.203052	-0.121605
C	1.804033	1.208321	0.006301
C	1.096377	-0.000031	0.071494
C	-0.320378	-0.000123	0.203338
C	-1.532040	-0.000203	0.280411
C	-4.153826	-0.000266	0.336174
C	-2.880286	-0.000425	0.554143
C	-5.206437	0.000503	-0.712298
H	1.259602	-2.143582	0.056105
H	3.721282	-2.143284	-0.171941
H	4.958408	0.000194	-0.286240
H	3.721097	2.143527	-0.171269
H	1.259417	2.143541	0.056772
H	-3.644405	-0.001205	1.563047
H	-5.842788	-0.882136	-0.617451
H	-4.745810	0.001555	-1.707342
H	-5.843201	0.882660	-0.615768

Frequencies

-2181.8281	28.5883	52.4793
65.3579	100.9567	132.5854
211.0117	250.3775	267.6215
352.6243	399.6159	413.2537
485.7005	497.8151	549.7508
571.3613	643.2712	669.0747
712.6381	781.6361	839.7466
867.0333	945.1339	1001.6954
1021.0860	1021.9389	1025.3476
1031.2476	1055.1614	1073.4513
1112.6710	1188.1572	1209.4862
1313.3459	1316.7091	1356.5937
1389.3401	1469.0178	1477.8808
1486.9154	1539.3779	1642.7080
1673.8575	1968.0383	2269.9420
2387.2351	3022.0361	3110.0044
3130.0497	3188.2509	3196.9307
3207.5168	3214.7274	3220.1890

i8 – i9

Cartesian coordinates

C	-1.514941	-1.210118	0.106237
C	-2.863715	-1.203072	-0.207620
C	-3.545294	0.000050	-0.365889
C	-2.863724	1.203130	-0.207292
C	-1.514950	1.210090	0.106568
C	-0.816810	-0.000034	0.267801
C	0.557719	-0.000077	0.590516
C	1.777944	-0.000099	0.735578
C	3.273960	0.000066	-0.364658

C	3.200064	-0.000224	1.089343
C	3.939974	0.000267	-1.488329
H	-0.983416	-2.146256	0.228915
H	-3.388699	-2.143669	-0.330738
H	-4.600954	0.000079	-0.611104
H	-3.388709	2.143762	-0.330146
H	-0.983427	2.146193	0.229512
H	3.547382	0.902638	1.597462
H	3.547295	-0.903325	1.597099
H	3.436972	0.000468	-2.451219
H	5.032186	0.000237	-1.503303

Frequencies

-682.3928	35.9979	51.8581
93.5088	154.1448	156.3072
265.3577	286.6048	363.4456
407.1236	413.4358	510.3382
517.9624	551.4116	640.9749
708.0890	710.2620	773.7841
860.1168	901.3538	922.4600
932.5143	939.1022	997.5189
998.7716	1015.4155	1017.1793
1031.4137	1060.3903	1112.5042
1135.7418	1186.1124	1201.6271
1209.4610	1311.6925	1314.1068
1355.5234	1430.0692	1449.4080
1484.6793	1531.4459	1631.6208
1657.3771	1792.0617	2132.2734
3051.8126	3075.0528	3117.7747
3180.2285	3186.7476	3194.4942

3206.4691	3212.7899	3218.8703

i9 – i11

Cartesian coordinates

C	-1.915443	-1.196689	0.058573
C	-3.270646	-0.912805	0.037541
C	-3.715829	0.404995	-0.006422
C	-2.789463	1.443217	-0.030828
C	-1.431186	1.174082	-0.014633
C	-0.971878	-0.154427	0.029351
C	0.411453	-0.433299	0.051203
C	1.610323	-0.674056	-0.080764
C	3.030468	-0.916701	-0.051216
C	3.964493	0.033844	0.233779
C	4.788163	1.017874	0.017419
H	-1.569449	-2.222720	0.097770
H	-3.986806	-1.726506	0.058011
H	-4.777592	0.621399	-0.020151
H	-3.129224	2.472221	-0.064124
H	-0.709831	1.982283	-0.034077
H	2.283091	-0.783899	-1.247793
H	3.327139	-1.961317	-0.089676
H	4.941985	1.423493	-0.984476
H	5.357957	1.478832	0.820487

Frequencies

-1920.7034	42.9513	47.5135
81.0381	126.0104	216.8519
242.0408	307.8458	352.0003
412.5413	419.5903	510.5105

543.1810	559.5891	629.9522
641.0079	708.5958	715.2190
753.1107	773.2001	859.6402
918.2231	931.6344	956.0333
993.8509	997.3058	1009.6131
1015.7517	1017.1841	1056.9394
1099.4758	1112.9746	1186.5640
1207.1254	1301.4155	1314.4312
1356.1879	1367.5915	1457.0023
1484.9177	1531.2742	1607.2646
1631.8933	1666.3496	1912.6153
2179.5837	3079.9651	3165.7478
3170.5114	3187.6687	3195.0192
3206.9607	3212.7617	3219.4007

i9 – i10

Cartesian coordinates

C	-1.894672	-1.192517	-0.123355
C	-3.253697	-0.925081	-0.197672
C	-3.717053	0.381214	-0.090813
C	-2.813886	1.422219	0.091598
C	-1.453663	1.161829	0.167846
C	-0.979981	-0.150209	0.060796
C	0.423938	-0.420597	0.138516
C	1.603205	-0.642492	0.204738
C	3.037281	-0.906920	0.287115
C	3.870237	0.159639	-0.361407
C	4.813179	1.021460	-0.193889
H	-1.528867	-2.208978	-0.206939
H	-3.954131	-1.740048	-0.340218

H	-4.779593	0.587519	-0.149657
H	-3.170444	2.442495	0.175177
H	-0.745431	1.969354	0.310029
H	3.258189	-1.868082	-0.187706
H	3.340524	-0.989614	1.339825
H	4.251986	0.890455	-1.348120
H	5.518435	1.465625	0.506765

Frequencies

-2108.5527	7.8376	37.0519
77.5536	90.5746	165.6452
231.1406	262.8435	337.5420
357.4406	406.4152	414.8211
500.4291	553.9903	568.9864
642.3671	650.1868	715.4673
745.1486	786.2434	827.8078
871.0174	903.5735	937.6845
951.1341	1003.9131	1011.7405
1023.4877	1024.5684	1064.0545
1111.6680	1188.6064	1208.9079
1217.8865	1281.9742	1318.6774
1327.8673	1355.6957	1455.6048
1488.2765	1542.7230	1648.7873
1681.0546	1875.6236	2383.9460
2440.7925	3013.0827	3080.1600
3120.0504	3187.8020	3196.8649
3206.8045	3214.2766	3219.8149

i4 – p4

Cartesian coordinates

C	-1.443836	1.142503	0.000012
C	-2.822817	1.287326	0.000010
C	-3.643897	0.165263	-0.000001
C	-3.081017	-1.106097	-0.000011
C	-1.702907	-1.259490	-0.000010
C	-0.870225	-0.134373	0.000002
C	0.549448	-0.286143	0.000004
C	1.747776	-0.423899	0.000005
C	3.128097	-0.548182	0.000006
C	4.167297	-1.204028	0.000014
C	3.590213	1.644236	-0.000021
H	-0.798591	2.012898	0.000021
H	-3.258640	2.279715	0.000018
H	-4.721586	0.281510	-0.000003
H	-3.718636	-1.982572	-0.000021
H	-1.257699	-2.247110	-0.000018
H	5.185100	-1.517063	0.000017
H	3.094124	1.917577	-0.923830
H	4.673016	1.674752	-0.000024
H	3.094128	1.917599	0.923784

Frequencies

-605.2552	11.5381	56.6844
73.1724	97.7950	143.1082
201.0713	248.2612	365.1806
385.3750	414.0095	424.6704
477.8417	504.4992	550.4921
562.3710	568.8506	580.8465
642.9322	682.6263	708.2390

714.1968	787.3522	871.1903
897.0402	953.9580	978.6770
1005.4877	1024.4666	1025.9854
1063.4258	1112.9531	1189.7066
1210.0226	1299.2615	1319.6676
1356.8090	1419.2791	1423.3337
1488.5005	1541.6115	1647.7871
1679.1863	2001.5670	2360.6580
3096.2310	3189.5556	3198.6665
3208.0185	3215.7825	3221.1531
3265.0428	3265.5492	3453.5765

i4 – i7

Cartesian coordinates

C	-1.606843	1.204962	-0.000090
C	-2.993164	1.233180	-0.000205
C	-3.717478	0.046303	-0.000121
C	-3.049476	-1.173125	0.000087
C	-1.663350	-1.209687	0.000205
C	-0.927401	-0.018929	0.000114
C	0.500265	-0.053272	0.000179
C	1.705803	-0.091886	0.000165
C	3.120473	-0.105812	0.000034
C	4.018555	-1.084425	-0.000439
C	4.077987	1.056115	0.000193
H	-1.037065	2.126474	-0.000144
H	-3.510805	2.185515	-0.000357
H	-4.801125	0.071718	-0.000223
H	-3.611064	-2.100215	0.000136
H	-1.137018	-2.156643	0.000346

H	4.008900	-2.171299	-0.000911
H	4.173954	1.628547	-0.921618
H	4.947438	-0.032531	-0.000297
H	4.174562	1.627888	0.922334

Frequencies

-2203.4688	22.0670	61.3625
70.5124	177.2740	196.8588
320.4878	367.3529	414.1151
462.7533	479.8975	502.2329
569.8668	579.4511	603.1084
643.9494	714.0500	726.8499
752.2775	786.8941	870.2610
904.6833	952.4537	970.1459
997.3039	1004.4135	1024.6219
1024.7649	1053.9983	1062.1614
1112.7026	1114.8911	1189.2419
1196.4306	1211.2627	1319.8592
1331.1019	1356.3308	1425.7867
1488.6288	1541.1932	1647.8490
1673.2550	1693.5308	1909.5724
2351.9153	3087.5542	3175.2161
3189.3326	3191.7621	3198.5662
3208.1131	3215.8357	3221.1051

i10 – p1

Cartesian coordinates

C	-1.449600	1.140613	-0.252572
C	-2.809149	1.389761	-0.137646
C	-3.688977	0.353481	0.153643

C	-3.203474	-0.937004	0.330920
C	-1.844977	-1.193569	0.218596
C	-0.954552	-0.155650	-0.074900
C	0.448840	-0.412887	-0.192955
C	1.628507	-0.614942	-0.294343
C	3.062659	-0.869875	-0.422427
C	3.902387	0.228915	0.091794
C	4.704339	0.872253	0.728935
H	-0.758731	1.943600	-0.479962
H	-3.183506	2.397501	-0.276568
H	-4.751055	0.551268	0.242382
H	-3.885905	-1.747916	0.558352
H	-1.461658	-2.197536	0.356828
H	3.309883	-1.048968	-1.473519
H	3.324298	-1.779879	0.127329
H	3.257262	1.528489	-1.156809
H	5.373390	1.546871	1.207695

Frequencies

-799.1961	9.6084	50.1261
80.4317	111.6457	159.5522
251.1485	332.2560	364.5403
401.5106	411.8295	414.3977
495.4590	539.9982	555.6770
599.5996	642.7852	662.9925
714.0960	715.2369	745.2249
786.0467	871.1207	932.8881
937.3593	951.7159	1004.1893
1021.7584	1023.9174	1024.8127
1064.6314	1112.0789	1188.7165

1209.2707	1244.8925	1290.1644
1319.3354	1355.9097	1356.3957
1461.9256	1488.5260	1542.9584
1649.1831	1681.1969	2157.8488
2388.8808	3052.7459	3092.1799
3188.0466	3197.2066	3206.9922
3214.6037	3220.0240	3475.0155

i10 – p6

Cartesian coordinates

C	1.702535	-1.201534	0.024021
C	3.053718	-1.142682	0.327156
C	3.705232	0.084493	0.391434
C	2.996861	1.256954	0.150260
C	1.645366	1.207485	-0.153188
C	0.980004	-0.024967	-0.220507
C	-0.407917	-0.080096	-0.529865
C	-1.585316	-0.123722	-0.824225
C	-2.965180	-0.176398	-1.040055
C	-3.857680	-0.132152	0.888812
C	-4.994329	0.329313	0.940577
H	1.191248	-2.155391	-0.028421
H	3.602126	-2.058922	0.513890
H	4.762016	0.127001	0.628659
H	3.500876	2.215431	0.199216
H	1.089257	2.118074	-0.341372
H	-3.389002	-1.128680	-1.339611
H	-3.437311	0.698728	-1.470406
H	-3.020601	-0.563109	1.402070
H	-5.938372	0.766703	0.709453

Frequencies

-726.7527	25.5515	34.6171
51.5243	95.6689	126.8817
256.3877	297.0050	306.9050
414.3534	420.7683	464.3495
511.5085	542.1874	551.3096
590.5809	641.9397	702.6585
712.0249	734.1064	781.5493
782.9170	827.6455	867.4210
938.7292	947.0262	1002.5204
1021.3958	1021.7477	1037.5396
1066.4465	1072.1047	1112.2240
1188.1566	1208.4307	1317.6558
1329.8770	1355.5391	1462.4673
1487.1935	1541.6042	1642.1070
1672.4637	1853.0867	2226.0252
3148.1013	3188.0909	3196.8782
3207.1128	3214.4227	3220.0285
3237.9009	3333.6698	3454.0779

i1 – i2

Cartesian coordinates

C	-1.976718	-1.192727	0.058843
C	-3.332361	-0.901395	0.084254
C	-3.766952	0.418378	0.033694
C	-2.836913	1.448884	-0.042478
C	-1.479858	1.165228	-0.067848
C	-1.033829	-0.160970	-0.017646
C	0.364676	-0.452304	-0.044854

C	1.548608	-0.679517	-0.069034
C	2.951944	-0.970498	-0.101881
H	-1.633689	-2.219787	0.099086
H	-4.054066	-1.708149	0.143558
H	-4.827411	0.642217	0.053890
H	-3.170625	2.479591	-0.083244
H	-0.750781	1.964496	-0.127099
H	3.207277	-2.029244	-0.223839
C	3.887467	-0.063082	0.003838
C	4.935005	0.930350	0.111831
H	4.668631	1.856402	-0.412912
H	5.888946	0.578387	-0.304932
H	5.105289	1.182007	1.163178

Frequencies

-274.3866	26.8231	57.0113
91.6697	105.1958	165.4290
253.2449	298.7281	360.2125
409.4053	414.0087	498.7024
553.9845	580.0321	642.9791
711.0477	729.0353	780.6338
798.8620	866.4032	874.6019
943.1507	984.3381	1000.1767
1016.7123	1023.0968	1029.5733
1041.9743	1064.6794	1111.3534
1188.2528	1206.9702	1287.6745
1316.1250	1323.5161	1355.1953
1406.9552	1458.0537	1474.8654
1487.6731	1541.1858	1646.3186
1678.8494	1833.0424	2348.8947

2993.0683	3041.5145	3048.2608
3100.4989	3191.3199	3200.7905
3210.7610	3218.8282	3224.0112

i4 – i6

Cartesian coordinates

C	1.552349	-1.180951	-0.054020
C	2.933156	-1.279512	-0.026667
C	3.722810	-0.134883	0.029957
C	3.115651	1.117210	0.059919
C	1.736095	1.231695	0.034440
C	0.929397	0.080257	-0.023330
C	-0.475694	0.189519	-0.050845
C	-1.698398	0.294681	-0.054903
C	-3.033495	0.398479	-0.151454
C	-4.260614	0.832021	0.130061
H	0.939239	-2.073256	-0.098693
H	3.398617	-2.258474	-0.050109
H	4.803081	-0.217919	0.050734
H	3.724055	2.013523	0.104396
H	1.264345	2.206658	0.059246
H	-4.961493	1.039337	-0.688512
C	-3.990374	-1.001551	0.136379
H	-3.516772	-1.603045	-0.641961
H	-5.064664	-1.040775	-0.052392
H	-3.771693	-1.347838	1.140070

Frequencies

-435.4001	52.0191	58.4668
66.5085	127.8973	176.6401

231.8169	259.5221	356.5747
378.2749	412.0515	469.7127
518.2276	582.1478	611.1792
641.8041	693.0273	707.8858
771.9393	856.3494	861.8384
881.3647	918.4216	927.7068
972.5062	995.6738	1010.5002
1013.8397	1018.5906	1058.5564
1112.9220	1185.6933	1206.1753
1289.6734	1313.2189	1316.1832
1355.8697	1443.4421	1469.5784
1484.0366	1527.4076	1629.3433
1656.1306	1789.2307	2145.1553
3034.8349	3067.7253	3141.3799
3186.8004	3194.0568	3206.3217
3212.3755	3218.9694	3219.2821

i5 – i9

Cartesian coordinates

C	-1.925068	-1.199963	0.055221
C	-3.292082	-0.971253	0.090271
C	-3.786761	0.327743	0.052257
C	-2.905656	1.401113	-0.018968
C	-1.536992	1.181645	-0.050363
C	-1.031325	-0.123994	-0.014709
C	0.376184	-0.353302	-0.049217
C	1.569304	-0.537269	-0.073345
C	2.966674	-0.776585	-0.111888
C	3.926854	0.288553	-0.204186
C	5.080741	0.676096	0.302476

H	-1.535606	-2.210674	0.081936
H	-3.975127	-1.811149	0.145789
H	-4.856075	0.503141	0.078189
H	-3.286663	2.415517	-0.048240
H	-0.846305	2.014741	-0.102534
H	3.408919	-0.335071	-1.264585
H	3.316739	-1.771840	0.164318
H	5.752185	1.339107	-0.231981
H	5.370691	0.379515	1.311818

Frequencies

-1989.3701	30.6000	50.9735
86.5090	131.4059	194.8547
279.5605	286.6433	367.9159
412.6729	414.2815	506.7558
547.9830	570.8874	642.5115
659.4943	713.7956	744.9228
783.6939	832.0746	869.0260
891.5026	948.3639	949.3788
1003.1065	1022.7496	1023.3441
1029.3058	1061.4475	1099.8268
1112.6948	1129.6298	1188.7872
1209.1542	1259.2941	1318.8404
1355.4363	1356.5573	1442.4128
1488.2674	1543.4459	1645.7337
1678.0547	1725.0717	2169.8099
2319.5301	3090.1503	3109.8195
3188.5858	3197.2431	3206.3231
3207.5322	3214.6525	3220.3219

i11 – p5

Cartesian coordinates

C	1.674344	-1.210402	0.192382
C	2.976543	-1.203094	-0.280759
C	3.630789	0.001792	-0.517431
C	2.974967	1.205275	-0.277989
C	1.672762	1.209789	0.195166
C	1.004465	-0.001021	0.435184
C	-0.330917	-0.002390	0.912938
C	-1.537988	-0.003123	1.111981
C	-3.904508	0.000857	-0.586525
C	-2.665023	-0.000271	-0.778126
C	-5.189695	0.001859	-0.231407
H	1.159967	-2.145487	0.379021
H	3.484936	-2.142238	-0.466433
H	4.649699	0.002882	-0.886958
H	3.482127	2.145507	-0.461513
H	1.157152	2.143765	0.383940
H	-2.413038	-0.004355	1.724574
H	-1.892786	-0.000314	-1.519702
H	-5.730255	0.932448	-0.101744
H	-5.732237	-0.927841	-0.103653

Frequencies

-651.7144	8.9490	40.3977
41.1842	111.3223	168.0384
173.2083	311.2186	381.7158
384.9194	413.3621	464.6560
498.4611	543.2384	546.9998
640.2902	706.7695	710.5324

716.5527	724.3264	781.6038
784.6837	792.5046	829.7917
867.1773	947.1873	1003.6314
1020.8439	1023.0963	1032.7515
1058.6717	1112.7645	1157.3002
1188.4728	1204.7912	1234.9091
1318.5646	1355.8080	1467.2704
1486.3700	1531.0581	1639.0081
1666.2865	1900.6253	2079.1819
3151.1119	3189.5446	3198.4227
3208.4524	3215.8195	3221.1244
3242.4129	3365.0484	3402.4058

i11 – p2

Cartesian coordinates

C	-1.717429	1.208435	-0.032296
C	-3.092936	1.203905	0.140765
C	-3.783781	0.000152	0.227548
C	-3.093072	-1.203707	0.141158
C	-1.717566	-1.208447	-0.031898
C	-1.014655	-0.000061	-0.120671
C	0.399336	-0.000173	-0.296166
C	1.613641	-0.000221	-0.329328
C	4.033016	-0.000019	-0.021835
C	2.970952	-0.000476	-0.787645
C	5.107651	0.000410	0.705215
H	-1.172908	2.142638	-0.098738
H	-3.628481	2.143752	0.209482
H	-4.859208	0.000235	0.363162
H	-3.628721	-2.143472	0.210181

H	-1.173148	-2.142733	-0.098032
H	1.990258	0.000690	1.660947
H	3.109880	-0.001101	-1.866578
H	5.565697	-0.929675	1.025783
H	5.565685	0.930872	1.024708

Frequencies

-693.7088	11.7570	58.5581
72.5969	101.4947	156.9068
274.6070	288.7581	345.8883
346.3382	413.8379	428.4074
461.6006	534.2884	566.3000
612.8166	636.9236	652.8434
712.8473	771.4898	785.8857
869.6565	889.6279	895.8787
952.1045	979.5906	1004.5922
1008.6897	1024.0123	1024.8106
1061.8046	1112.6666	1140.0295
1189.2960	1209.1507	1277.7992
1318.7637	1356.3310	1372.8725
1478.3834	1487.6531	1542.1557
1645.4350	1675.9173	2083.1958
2274.6818	3143.5102	3152.0372
3189.2654	3198.5754	3208.3577
3216.1240	3221.1683	3227.4364

i6 – p3

Cartesian coordinates

C	1.856097	-1.208729	-0.008190
C	3.242438	-1.203866	-0.005167

C	3.938321	-0.000003	-0.003627
C	3.242451	1.203867	-0.004961
C	1.856110	1.208745	-0.007980
C	1.149047	0.000012	-0.009817
C	-0.276568	0.000019	-0.012560
C	-1.485738	0.000023	-0.015111
C	-4.061489	-0.000052	0.059159
C	-2.849360	0.000030	-0.023033
C	-5.501797	0.000174	-0.185088
H	1.307730	-2.143024	-0.008854
H	3.782406	-2.143631	-0.003715
H	5.022235	-0.000009	-0.001048
H	3.782428	2.143626	-0.003348
H	1.307753	2.143047	-0.008483
H	-4.226991	-0.002032	2.074117
H	-5.968499	-0.883910	0.254160
H	-5.695581	0.001327	-1.260650
H	-5.968554	0.883278	0.256066

Frequencies

-707.5136	37.0678	52.6330
54.1285	142.9519	146.6215
173.0080	296.1818	326.7254
337.3872	412.9692	417.1935
437.1122	493.3258	525.4455
573.6207	584.5919	644.4575
658.9445	713.1712	786.8386
841.8571	870.0453	953.4053
1005.0036	1024.4127	1025.5801
1044.2889	1060.3974	1061.0999

1113.2095	1135.8082	1189.6102
1210.5805	1319.6665	1351.6082
1357.0261	1417.4024	1478.3409
1481.6184	1488.0398	1543.2950
1646.7865	1677.9786	2251.5566
2348.0377	3051.5746	3129.8284
3131.9435	3190.3606	3199.9263
3209.1299	3217.1653	3222.0890

i6 – p4

Cartesian coordinates

C	-1.780948	-1.208942	-0.021739
C	-3.149604	-1.202823	0.198319
C	-3.836638	0.001376	0.305893
C	-3.148900	1.204624	0.192370
C	-1.780240	1.208866	-0.027717
C	-1.082048	-0.000521	-0.137067
C	0.324369	-0.001525	-0.362874
C	1.520080	-0.002438	-0.553145
C	4.079743	-0.003907	-0.836903
C	2.862383	-0.003531	-0.777434
C	5.049949	0.005870	1.347514
H	-1.240273	-2.143857	-0.106640
H	-3.682834	-2.142329	0.286209
H	-4.906780	0.002115	0.477934
H	-3.681586	2.144861	0.275633
H	-1.239026	2.143040	-0.117219
H	5.058313	-0.005804	-1.258099
H	5.603100	-0.922698	1.277684
H	4.139467	0.007177	1.931340

H	5.600743	0.935210	1.269854

Frequencies

-443.0206	18.6053	36.0442
44.8013	72.5929	101.4350
225.6967	228.1727	365.7478
375.8399	412.8652	424.4724
463.4472	488.8626	489.6720
562.2044	586.7314	644.0597
667.0570	675.1382	708.9962
712.9384	785.2326	799.8216
869.6558	951.7784	987.8256
1004.7782	1023.8379	1025.2035
1063.5990	1113.6271	1189.9009
1210.2064	1309.7381	1318.9669
1357.4948	1411.7979	1420.5758
1488.1004	1540.4260	1645.5709
1676.1958	2086.5222	2303.3201
3103.1510	3190.3061	3199.2282
3209.0516	3216.4406	3221.6902
3272.8347	3282.9784	3441.6135

i2 – i5

Cartesian coordinates

C	1.923612	-1.205689	0.057565
C	3.293824	-1.001895	0.114196
C	3.813366	0.287538	0.079524
C	2.952910	1.376054	-0.012100
C	1.581369	1.180912	-0.068191
C	1.049350	-0.114759	-0.034024

C	-0.360318	-0.318724	-0.092225
C	-1.557261	-0.480771	-0.146469
C	-2.950146	-0.698076	-0.177169
C	-3.873396	0.287591	-0.238259
C	-5.165558	0.466413	0.283714
H	1.515166	-2.208922	0.083154
H	3.960236	-1.854044	0.184972
H	4.885147	0.443527	0.123267
H	3.352783	2.383321	-0.040245
H	0.907262	2.026062	-0.140367
H	-3.292833	-1.734050	-0.214693
H	-4.147248	1.281862	0.549094
H	-5.594669	-0.191690	1.043465
H	-5.832354	1.182373	-0.188015

Frequencies

-1791.2439	34.4810	53.9851
86.1791	147.3993	174.5075
247.5352	323.6010	369.7862
414.6898	423.0386	510.9616
544.7915	563.9981	611.0216
644.0205	713.6339	758.0844
783.4907	796.4811	868.1726
919.5920	946.6072	988.7368
1001.9387	1020.8284	1023.6130
1053.8848	1067.1737	1112.0308
1126.1317	1175.3550	1188.0505
1208.8748	1277.5388	1318.0868
1355.2626	1356.1337	1476.1591
1487.8478	1539.7378	1587.2215

1645.0804	1679.2298	2229.0520
2306.7578	3061.4716	3099.3611
3187.2184	3196.1118	3196.4062
3206.7546	3214.1403	3219.5875

i2 – i3

Cartesian coordinates

C	-1.560924	-1.209637	0.097517
C	-2.905963	-1.203995	-0.230334
C	-3.586073	-0.000559	-0.397362
C	-2.906219	1.203332	-0.232519
C	-1.561178	1.209870	0.095306
C	-0.858852	0.000346	0.265727
C	0.508895	0.000804	0.603650
C	1.737357	0.001013	0.765530
C	3.296028	-0.000138	-0.151619
C	3.112129	0.001434	1.136948
C	4.176263	-0.001645	-1.325701
H	-1.030120	-2.145144	0.228953
H	-3.430466	-2.144553	-0.357096
H	-4.639335	-0.000904	-0.653281
H	-3.430933	2.143537	-0.360991
H	-1.030576	2.145735	0.225022
H	3.654333	0.002519	2.076841
H	3.982353	0.878156	-1.944953
H	3.982286	-0.882984	-1.942738
H	5.233680	-0.001316	-1.034619

Frequencies

-679.0092	45.8815	50.7985
88.1999	122.1286	153.3255
224.5194	270.9990	374.6082
379.0548	415.8667	423.7518
518.1443	520.0761	639.1815
706.7820	718.6032	775.2077
788.8420	859.5181	881.4841
933.4336	997.5612	1000.3745
1014.0306	1016.7601	1034.9622
1045.1187	1059.0076	1110.1666
1159.4066	1184.5388	1204.2027
1310.6692	1343.2158	1352.6973
1397.9774	1467.7985	1472.8092
1482.4988	1528.7779	1626.3833
1650.4622	1827.5307	2055.5475
3018.1909	3099.1583	3117.7782
3171.7487	3184.5839	3192.4235
3204.9988	3211.4527	3217.4367

i3 – i4

Cartesian coordinates

C	-1.558989	1.209962	0.037522
C	-2.943409	1.203937	0.054588
C	-3.642516	0.000000	0.063290
C	-2.943397	-1.203932	0.054754
C	-1.558977	-1.209946	0.037691
C	-0.838786	0.000011	0.027291
C	0.571136	0.000015	0.014882
C	1.801146	-0.000010	-0.121353
C	3.229574	-0.000021	-0.090320

C	3.101795	-0.000190	-1.385954
C	4.232115	0.000135	1.009342
H	-1.012071	2.145320	0.030357
H	-3.483528	2.144068	0.060600
H	-4.726332	-0.000004	0.076938
H	-3.483507	-2.144067	0.060898
H	-1.012049	-2.145300	0.030661
H	3.580638	-0.000311	-2.353773
H	4.096928	-0.882151	1.639828
H	4.096930	0.882608	1.639567
H	5.244835	0.000074	0.604535

Frequencies

-722.9327	50.6738	58.6041
67.7465	135.7700	180.3339
244.6581	275.6660	373.8390
414.7592	446.6317	454.6945
520.0755	530.1599	640.0133
664.8101	691.0645	706.9830
734.5903	776.3937	846.9447
860.5350	936.3747	998.4557
998.9195	1015.6568	1017.2753
1049.5015	1057.6061	1110.6317
1157.6980	1184.9082	1204.8956
1312.2515	1349.6206	1353.1430
1406.8458	1479.5289	1483.0818
1487.7390	1529.9748	1628.7090
1652.8078	1787.4037	2065.3843
3053.0567	3126.2278	3150.6191
3186.0206	3194.2548	3206.0432

3212.9681	3218.6151	3256.3367

i5 – p2

Cartesian coordinates

C	-1.926298	-1.198641	-0.038036
C	-3.287074	-0.932869	-0.033338
C	-3.744231	0.379791	0.002639
C	-2.833027	1.429588	0.033651
C	-1.470560	1.172509	0.028239
C	-1.002848	-0.146739	-0.007595
C	0.398986	-0.414130	-0.012374
C	1.586520	-0.626048	-0.017392
C	2.973988	-0.908896	-0.026075
C	3.912670	0.026333	0.031087
C	4.818524	0.931143	-0.200821
H	-1.565002	-2.219710	-0.065037
H	-3.994277	-1.753977	-0.057238
H	-4.808697	0.584243	0.006746
H	-3.185539	2.454211	0.062146
H	-0.755829	1.986341	0.052345
H	3.278360	-1.952548	-0.018582
H	4.064865	-0.007176	2.092121
H	5.346378	1.442127	0.594582
H	5.059826	1.194243	-1.226980

Frequencies

-721.0973	25.1627	49.2824
76.8173	111.5851	145.5376
252.3135	340.4779	365.1202
376.4277	414.2592	416.8572

462.8120	542.9533	553.0114
623.5233	643.3646	654.7904
713.9022	762.1290	785.6282
869.5460	874.5931	913.7743
951.0508	990.1125	1004.0285
1008.9766	1023.8160	1024.2490
1061.3592	1112.5823	1119.1305
1189.2838	1209.2619	1292.3603
1319.4760	1356.4224	1380.3818
1471.0602	1488.3638	1544.3225
1646.7612	1679.2655	2007.1128
2337.7925	3143.5771	3159.1944
3189.1324	3197.8750	3208.0740
3215.2505	3220.7623	3237.1837

i5 – i11

Cartesian coordinates

C	-1.734587	-1.219336	0.088494
C	-3.097829	-1.124924	0.308945
C	-3.730278	0.115223	0.284930
C	-2.988140	1.266633	0.036747
C	-1.624489	1.185327	-0.187407
C	-0.972644	-0.062302	-0.161964
C	0.414327	-0.152388	-0.395062
C	1.660258	-0.164880	-0.359073
C	2.993784	-0.276555	-0.877279
C	3.779494	0.126960	0.129173
C	4.929756	0.217301	0.813171
H	-1.237752	-2.181938	0.107667
H	-3.673152	-2.023044	0.502651

H	-4.797927	0.183966	0.458450
H	-3.477889	2.233553	0.017681
H	-1.042936	2.078277	-0.382989
H	3.286423	-0.581810	-1.877729
H	2.424619	0.346558	0.777122
H	5.234460	1.151628	1.270046
H	5.506246	-0.673541	1.043049

Frequencies

-2250.0929	39.7291	53.0899
100.2441	162.7262	191.5489
214.5718	307.5308	349.2599
413.4028	431.4110	468.4195
533.1833	535.1236	592.6753
638.0921	705.8174	751.4579
775.6977	777.5623	827.5740
861.4441	902.1309	940.5231
983.8292	1000.7470	1013.9523
1017.8665	1019.0825	1050.3865
1059.4878	1111.7772	1186.2971
1202.0791	1243.2016	1314.1188
1353.7484	1356.2670	1451.2551
1483.0455	1522.8838	1535.5284
1629.0039	1655.0121	1839.5133
2074.4173	3141.7454	3177.3944
3188.4122	3196.7145	3207.6347
3214.6813	3220.1290	3235.0993

i5 – i10

Cartesian coordinates

C	-1.835619	-1.213339	-0.074797
C	-3.203634	-0.997814	-0.140738
C	-3.712584	0.295302	-0.090498
C	-2.845184	1.375886	0.026188
C	-1.475862	1.168835	0.092937
C	-0.955079	-0.130494	0.043244
C	0.453250	-0.347141	0.111229
C	1.645941	-0.531712	0.180529
C	3.054110	-0.724364	0.198544
C	3.969922	0.444420	0.465916
C	4.527657	0.544997	-0.723406
H	-1.434190	-2.218917	-0.113995
H	-3.876238	-1.843002	-0.232015
H	-4.782601	0.460561	-0.142410
H	-3.237687	2.385582	0.065247
H	-0.795636	2.007296	0.183253
H	3.394274	-1.692157	0.567486
H	4.090031	1.008155	1.384001
H	5.258166	1.170758	-1.227721
H	3.646374	-0.585729	-1.018726

Frequencies

-2222.2994	29.1469	56.8008
80.0054	145.1231	195.3601
308.3576	368.5721	414.3843
429.4283	494.8295	552.4394
575.1986	632.8415	643.4778
693.2855	713.4331	754.2633
784.3649	868.9577	888.4861

910.4602	941.2638	949.1038
992.6755	1003.1470	1022.6023
1024.0128	1058.6928	1084.0836
1112.3215	1161.9491	1188.5990
1208.7257	1211.8223	1274.9888
1318.7780	1355.9940	1364.5689
1488.0360	1542.7668	1645.6388
1657.3098	1677.3592	1846.5107
2300.6986	3122.6526	3173.0545
3188.3529	3197.3784	3207.4329
3214.9608	3215.9822	3220.3465

i5 – i6

Cartesian coordinates

C	-1.772519	1.208228	-0.007234
C	-3.158773	1.203419	-0.026415
C	-3.855439	0.000005	-0.035976
C	-3.158787	-1.203416	-0.026292
C	-1.772532	-1.208240	-0.007108
C	-1.063772	-0.000010	0.002695
C	0.361591	-0.000016	0.021445
C	1.572926	-0.000007	0.066245
C	4.077744	0.000254	0.662668
C	2.955995	-0.000038	-0.041126
C	5.067912	-0.000147	-0.463523
H	-1.224730	2.142891	0.000258
H	-3.698325	2.143508	-0.033976
H	-4.939250	0.000011	-0.050994
H	-3.698350	-2.143499	-0.033763
H	-1.224754	-2.142909	0.000473

H	4.243504	0.000665	1.733917
H	5.612195	0.920218	-0.673939
H	3.791405	-0.000432	-1.141003
H	5.612236	-0.920644	-0.673250

Frequencies

-2288.0483	21.1772	56.1196
56.7317	161.3893	172.2241
321.0868	335.9445	370.7831
413.0876	455.7006	483.6954
558.2998	565.8698	608.6530
643.4773	713.1406	771.7362
784.2936	841.2763	869.0039
916.7649	950.1269	1003.5742
1011.8084	1023.5829	1024.5147
1051.8364	1053.5503	1063.2768
1109.8019	1112.7032	1188.8203
1209.2149	1228.8052	1313.8009
1318.0933	1356.4921	1431.9487
1487.4135	1538.9743	1645.3172
1675.1033	1770.2436	1856.9022
2278.3453	3085.2114	3183.9951
3189.2602	3198.5082	3205.6201
3208.2754	3216.0419	3221.1560

i10 – i12

Cartesian coordinates

C	-1.646877	-1.210450	0.047399
C	-3.012641	-1.204004	-0.181932
C	-3.700996	0.000059	-0.297274

C	-3.012579	1.204076	-0.181829
C	-1.646815	1.210428	0.047499
C	-0.942046	-0.000034	0.164041
C	0.451648	-0.000068	0.402802
C	1.685716	-0.000074	0.383351
C	3.057053	-0.000069	0.965787
C	3.830047	0.000007	-0.310212
C	4.869889	0.000113	-0.979300
H	-1.107389	-2.145727	0.136960
H	-3.545137	-2.143879	-0.272448
H	-4.769892	0.000094	-0.476461
H	-3.545025	2.143989	-0.272259
H	-1.107275	2.145667	0.137143
H	3.255552	-0.886791	1.575974
H	3.255516	0.886594	1.576070
H	2.472709	-0.000054	-0.924307
H	5.496546	0.000204	-1.842646

Frequencies

-2255.4398	26.2760	54.9056
94.3029	169.8462	169.9903
293.9344	307.5772	339.3678
413.3893	447.3489	487.1479
493.9624	533.1988	567.4691
572.0553	641.3714	708.2654
721.8183	726.2731	778.0114
863.3625	879.9570	940.5909
967.2364	974.3179	1000.8460
1019.1630	1019.6656	1059.7771
1113.0441	1170.6337	1187.4943

1205.4204	1260.3081	1316.3118
1322.5590	1355.7081	1391.9692
1449.7877	1485.5473	1532.0873
1634.3367	1662.2626	2000.1138
2147.2424	3050.6757	3099.8438
3188.5586	3196.6806	3207.7803
3214.3500	3220.2229	3430.8105

i12 – p5

Cartesian coordinates

C	1.713555	-1.205724	0.152354
C	3.020407	-1.101584	-0.295232
C	3.603328	0.148572	-0.476975
C	2.870452	1.300077	-0.206905
C	1.562502	1.207961	0.240285
C	0.964956	-0.049252	0.423599
C	-0.375548	-0.148643	0.872813
C	-1.585442	-0.229701	1.049498
C	-2.710466	-0.285728	-0.855924
C	-4.025297	0.049238	-0.512108
C	-5.136277	0.339466	-0.131538
H	1.255555	-2.176958	0.296581
H	3.588046	-2.001141	-0.504190
H	4.626033	0.225839	-0.827526
H	3.322331	2.275428	-0.346268
H	0.988643	2.102042	0.453126
H	-2.455559	-0.289646	1.668767
H	-2.492460	-1.324768	-1.071983
H	-2.119110	0.448527	-1.388819
H	-6.126500	0.592585	0.161111

Frequencies

-624.7930	13.5692	40.2075
52.3274	110.4412	173.2238
175.7372	347.9194	381.7141
414.1272	445.3525	486.7450
540.4370	548.4217	565.2823
584.7257	637.7340	683.1990
710.1806	716.7138	747.6940
780.8767	784.2719	874.0841
882.3034	944.6485	1009.3395
1018.4287	1023.6786	1038.0934
1059.4759	1064.1057	1113.8096
1188.2252	1205.3151	1233.4730
1317.5632	1357.4568	1455.0527
1486.8782	1532.1226	1637.1195
1665.2084	2032.6059	2137.6356
3159.7298	3190.6758	3197.7263
3209.5637	3215.4262	3222.4977
3254.5092	3383.0400	3480.0267

i12 – p1

Cartesian coordinates

C	1.854030	-1.194573	0.087119
C	3.213536	-0.926348	0.130052
C	3.671897	0.383490	0.041259
C	2.763955	1.428501	-0.090793
C	1.402778	1.169517	-0.135180
C	0.934268	-0.147227	-0.046457
C	-0.465111	-0.415152	-0.089574

C	-1.655757	-0.623998	-0.021365
C	-3.068802	-0.916372	-0.308520
C	-3.964601	0.203402	-0.013795
C	-4.708938	1.112106	0.218047
H	1.490830	-2.212818	0.158080
H	3.918789	-1.742727	0.234740
H	4.735463	0.589714	0.075633
H	3.118199	2.450616	-0.158488
H	0.689665	1.978777	-0.236164
H	-1.905982	-0.452502	1.971279
H	-3.385695	-1.790950	0.267619
H	-3.159862	-1.181705	-1.367335
H	-5.364942	1.921519	0.429875

Frequencies

-689.6284	23.0838	48.2501
85.0566	116.4833	157.6844
256.2182	324.6405	330.9358
355.2944	413.9911	428.3914
448.5323	538.5524	565.9647
600.7306	642.3562	705.1301
713.1616	719.8800	750.6995
785.5135	869.9657	932.1685
939.4274	951.8726	1004.7306
1019.8476	1023.7054	1025.0856
1062.9538	1112.7375	1189.3266
1208.6441	1244.0041	1288.4819
1319.9997	1355.9825	1356.6337
1461.3324	1488.1188	1539.9990
1646.0137	1676.4197	2263.2885

2300.4897	3051.5598	3091.9426
3189.7547	3198.7967	3208.4905
3215.9988	3221.3020	3489.0484

Products

p1 + H

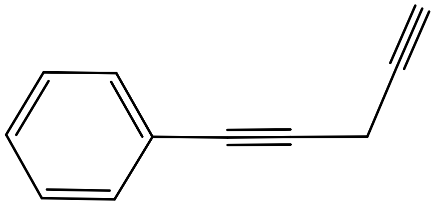

Cartesian coordinates

C	-1.846119	-1.203156	0.000044
C	-3.200494	-0.903304	0.000059
C	-3.624419	0.420657	0.000019
C	-2.686798	1.446975	-0.000036
C	-1.331121	1.154768	-0.000050
C	-0.897655	-0.175401	-0.000010
C	0.502793	-0.473583	-0.000027
C	1.681686	-0.703972	-0.000042
C	3.117619	-0.989096	-0.000066
C	3.945069	0.220378	0.000022
C	4.632033	1.201084	0.000092
H	-1.510917	-2.233610	0.000076
H	-3.928586	-1.706341	0.000101
H	-4.683606	0.651293	0.000030
H	-3.012864	2.480820	-0.000067
H	-0.595175	1.949803	-0.000092
H	3.369327	-1.594703	-0.877641
H	3.369331	-1.594835	0.877416
H	5.236930	2.075474	0.000154

Frequencies

14.2435	53.3202	85.6235
137.1190	234.6947	318.7034
328.2668	369.6740	395.2488
413.5478	535.0242	554.6790
601.7671	643.8025	707.2440
710.8468	711.6921	743.4454
780.8974	868.0859	928.1277
937.4402	945.5021	1002.3849
1018.7452	1021.5633	1024.5850
1064.0633	1112.2338	1188.9183
1208.0908	1251.0000	1289.7829
1317.7485	1355.9784	1361.0885
1454.6591	1488.6556	1542.5842
1648.5143	1680.6454	2262.9352
2389.0902	3044.4988	3079.1697
3191.9361	3202.6127	3210.4018
3221.9017	3226.6643	3486.5474

p2 + H

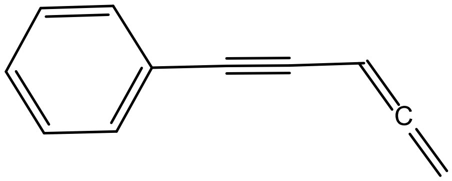

Cartesian coordinates

C	1.877822	-1.199389	0.000342
C	3.237989	-0.929493	0.000500
C	3.691643	0.384803	0.000212
C	2.776967	1.431783	-0.000240

C	1.415336	1.170150	-0.000399
C	0.951031	-0.150547	-0.000109
C	-0.451895	-0.419719	-0.000277
C	-1.638870	-0.626826	-0.000421
C	-3.034830	-0.906489	-0.000658
C	-3.961183	0.021635	0.000092
C	-4.887087	0.930526	0.000776
H	1.519401	-2.221960	0.000571
H	3.948154	-1.748486	0.000851
H	4.755663	0.591899	0.000345
H	3.126412	2.457994	-0.000471
H	0.697585	1.981706	-0.000748
H	-3.338783	-1.950446	-0.001566
H	-5.285039	1.324630	0.930709
H	-5.284928	1.326066	-0.928595

Frequencies

26.7109	49.9205	88.8027
139.0759	237.9870	330.4301
336.0206	365.9591	399.4731
413.3260	543.8834	549.3694
623.8778	642.4346	643.4021
710.6141	761.4234	780.2720
867.5865	888.7163	902.2869
944.2093	991.4467	1002.1595
1013.2792	1018.3907	1023.1961
1060.5246	1111.1730	1129.3156
1188.8983	1206.4720	1287.0623
1315.9380	1354.8771	1375.5031
1479.8937	1487.5551	1542.5475

1646.0316	1678.5926	2076.6635
2361.4123	3138.3061	3156.0261
3193.7019	3203.5975	3212.7761
3220.0924	3222.0732	3226.8927

p3 + H

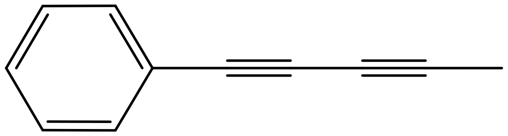

Cartesian coordinates

C	-1.801631	-1.208022	0.000003
C	-3.188224	-1.203515	-0.000008
C	-3.884558	-0.000067	-0.000014
C	-3.188311	1.203432	-0.000008
C	-1.801719	1.208040	0.000004
C	-1.094203	0.000035	0.000009
C	0.333053	0.000079	0.000017
C	1.540445	0.000086	0.000020
C	4.115565	-0.000003	0.000002
C	2.910496	0.000058	0.000015
C	5.569664	-0.000111	-0.000024
H	-1.253360	-2.142418	0.000007
H	-3.727872	-2.143544	-0.000013
H	-4.968502	-0.000107	-0.000023
H	-3.728028	2.143420	-0.000013
H	-1.253517	2.142476	0.000007
H	5.955539	1.022011	0.026206
H	5.956141	-0.488236	-0.898267
H	5.956133	-0.533661	0.871999

Frequencies

16.9328	52.6986	55.3850
149.0832	155.7200	284.3378
327.4723	334.1129	395.9178
413.4043	505.5932	532.8185
611.5241	631.4390	647.5555
654.4455	714.2006	789.1215
841.8620	870.2112	952.9443
1004.4438	1024.6992	1024.7157
1052.7943	1053.1280	1060.6799
1112.7545	1139.9673	1189.1987
1210.4287	1319.0986	1349.9609
1356.7799	1419.1093	1480.0281
1480.3628	1488.0679	1543.9077
1647.5547	1679.5289	2299.9413
2418.5996	3047.0589	3122.1467
3122.5703	3189.4553	3198.9831
3208.4682	3216.4707	3221.4423

p4 + methyl

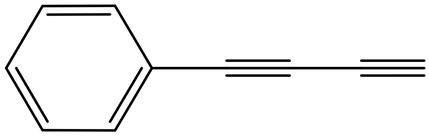

Cartesian coordinates

C	1.101948	-1.208695	0.000011
C	2.488454	-1.203815	0.000008
C	3.183998	-0.000052	-0.000007
C	2.488529	1.203755	-0.000018
C	1.102022	1.208726	-0.000015

C	0.396320	0.000039	-0.000001
C	-1.030822	0.000064	0.000002
C	-2.237708	0.000045	0.000005
C	-3.608004	0.000006	0.000007
C	-4.811060	-0.000041	0.000010
H	0.554126	-2.143332	0.000022
H	3.027906	-2.143845	0.000017
H	4.267994	-0.000084	-0.000009
H	3.028040	2.143752	-0.000029
H	0.554262	2.143399	-0.000024
H	-5.874388	-0.000083	0.000012

Frequencies

63.7994	69.2295	195.6241
221.8239	367.7979	374.7847
411.6634	488.2825	518.1404
596.0311	601.8844	643.1885
676.5256	688.1483	706.6563
710.1392	784.1149	867.8304
950.2029	984.6685	1003.3430
1021.2971	1022.5669	1063.3330
1113.0519	1188.8240	1213.3784
1307.8266	1319.5175	1358.0635
1487.3873	1542.1019	1646.0589
1677.6539	2201.1368	2388.4930
3197.9903	3206.9418	3216.0666
3223.5376	3229.0018	3483.4703

p5 + propargyl

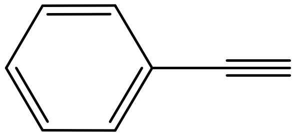

Cartesian coordinates

C	-0.119235	1.207139	-0.000010
C	-1.506283	1.203135	-0.000001
C	-2.202504	-0.000024	0.000006
C	-1.506242	-1.203162	0.000000
C	-0.119194	-1.207118	-0.000010
C	0.586875	0.000022	-0.000008
C	2.019321	0.000039	0.000009
C	3.220060	-0.000020	0.000012
H	0.428457	2.141945	-0.000012
H	-2.045318	2.143536	0.000006
H	-3.286553	-0.000045	0.000040
H	-2.045251	-2.143576	0.000007
H	0.428528	-2.141908	-0.000014
H	4.283350	-0.000015	-0.000019

Frequencies

137.3102	155.4248	366.7145
413.0878	474.7729	541.4990
555.6524	639.3903	663.7000
703.2868	711.5626	781.5994
782.0730	868.2462	948.3079
1002.3022	1019.5342	1023.1711
1060.8202	1112.1340	1187.9972
1211.5549	1234.3142	1319.7889
1357.2773	1487.8733	1539.4181
1648.1263	1679.1061	2244.4950

3196.3575	3205.2722	3214.7865
3222.1317	3227.8191	3483.1952

p6 + acetylene

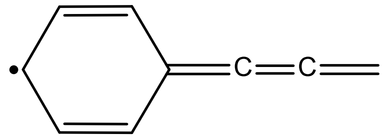

Cartesian coordinates

C	0.671604	-1.210796	-0.000006
C	2.056210	-1.204514	0.000006
C	2.753575	0.000022	0.000011
C	2.056174	1.204539	0.000005
C	0.671569	1.210779	-0.000006
C	-0.043767	-0.000019	-0.000012
C	-1.455605	-0.000030	-0.000021
C	-2.684676	-0.000009	-0.000010
C	-4.040349	0.000013	0.000018
H	0.124573	-2.145982	-0.000010
H	2.596376	-2.144351	0.000011
H	3.837522	0.000040	0.000018
H	2.596315	2.144390	0.000010
H	0.124511	2.145950	-0.000010
H	-4.593872	-0.931071	0.000029
H	-4.593840	0.931116	0.000039

Frequencies

85.2764	89.3696	217.5537
258.1128	311.6489	413.1716
413.5641	437.2621	531.7243
536.4907	637.7591	703.4181

713.6937	735.7243	774.9341
860.1720	938.0475	999.1616
1014.1072	1015.1412	1035.0325
1049.8153	1075.0877	1112.0165
1185.6922	1209.8008	1315.0182
1355.3024	1361.1603	1476.4679
1483.8384	1542.1545	1630.3313
1654.7317	2079.6909	3161.3898
3196.2121	3204.3989	3214.9694
3221.7924	3227.5904	3258.5642

Fragments

Methyl

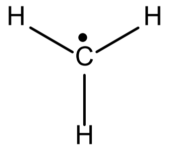

Cartesian coordinates

C	-0.000084	0.000054	0.000034
H	0.330345	-1.029327	-0.000069
H	-1.056667	0.228692	-0.000069
H	0.726826	0.800307	-0.000069

Frequencies

477.5911	1400.1589	1410.4961
3117.0568	3300.6263	3304.0767

Acetylene

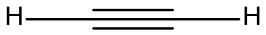

Cartesian coordinates

C	0.000000	0.000000	-0.598212
C	0.000000	0.000000	0.598211
H	0.000000	0.000000	-1.662213
H	0.000000	0.000000	1.662218

Frequencies

675.1654	675.1654	768.9824
768.9824	2088.1359	3423.0212
3530.5719		

Propargyl

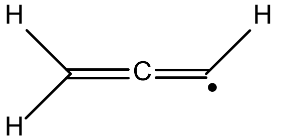

Cartesian coordinates

C	-0.117045	0.000056	-0.000045
C	-1.335904	-0.000010	-0.000022
C	1.251979	-0.000012	0.000022
H	-2.399234	-0.000053	0.000202
H	1.802428	-0.932378	0.000031
H	1.802624	0.932234	0.000031

Frequencies

355.7199	404.5134	494.3976
653.2122	674.3439	1036.2959
1088.1264	1459.5635	2025.8399
3166.2263	3267.3323	3471.5824

Supporting References

(1) Russo, M. V.; Lo Sterzo, C.; Franceschini, P.; Biagini, G.; Furlani, A. Synthesis of highly ethynylated mono and dinuclear Pt(II) tethers bearing the 4,4′-bis(ethynyl)biphenyl (debp) unit as central core. *J. Organomet. Chem.* **2001**, *619*, 49.
